# MFGE8 links absorption of dietary fatty acids with catabolism of enterocyte lipid stores through HNF4γ-dependent transcription of CES enzymes

**DOI:** 10.1016/j.celrep.2023.112249

**Published:** 2023-03-15

**Authors:** Ritwik Datta, Mohammad A. Gholampour, Christopher D. Yang, Regan Volk, Sinan Lin, Michael J. Podolsky, Thomas Arnold, Florian Rieder, Balyn W. Zaro, Michael Verzi, Richard Lehner, Nada Abumrad, Carlos O. Lizama, Kamran Atabai

**Affiliations:** 1 Cardiovascular Research Institute, University of California, San Francisco, San Francisco, CA 94158, USA; 2 Department of Gastroenterology, Hepatology and Nutrition, Digestive Diseases and Surgery Institute, Department of Inflammation and Immunity, Lerner Research Institute, Cleveland Clinic, Cleveland, OH 44106, USA; 3 Department of Pediatrics, University of California, San Francisco, San Francisco, CA 94158, USA; 4 Rutgers University, New Brunswick, NJ 08854, USA; 5 Department of Pediatrics, University of Alberta, Edmonton, AB T6G 1C9, Canada; 6 Washington University School of Medicine in St. Louis, St. Louis, MO 63110, USA; 7 Lung Biology Center, University of California, San Francisco, San Francisco, CA 94143, USA; 8 Lead contact

## Abstract

Enterocytes modulate the extent of postprandial lipemia by storing dietary fats in cytoplasmic lipid droplets (cLDs). We have previously shown that the integrin ligand MFGE8 links absorption of dietary fats with activation of triglyceride (TG) hydrolases that catabolize cLDs for chylomicron production. Here, we identify CES1D as the key hydrolase downstream of the MFGE8-αvβ5 integrin pathway that regulates catabolism of diet-derived cLDs. *Mfge8* knockout (KO) enterocytes have reduced CES1D transcript and protein levels and reduced protein levels of the transcription factor HNF4γ. Both *Ces1d* and *Hnf4*γ KO mice have decreased enterocyte TG hydrolase activity coupled with retention of TG in cLDs. Mechanistically, MFGE8-dependent fatty acid uptake through CD36 stabilizes HNF4γ protein level; HNF4γ then increases *Ces1d* transcription. Our work identifies a regulatory network that regulates the severity of postprandial lipemia by linking dietary fat absorption with protein stabilization of a transcription factor that increases expression of hydrolases responsible for catabolizing diet-derived cLDs.

## In brief

Enterocytes limit the severity of postprandial lipemia by storing lipids absorbed from the diet as part of cytoplasmic lipid droplets (cLDs). Datta et al. identify a homeostatic pathway in which the integrin ligand MFGE8 links dietary fat absorption with hydrolysis of cLDs through the nuclear receptor HNF4γ and CES1 hydrolases.

## Graphical abstract

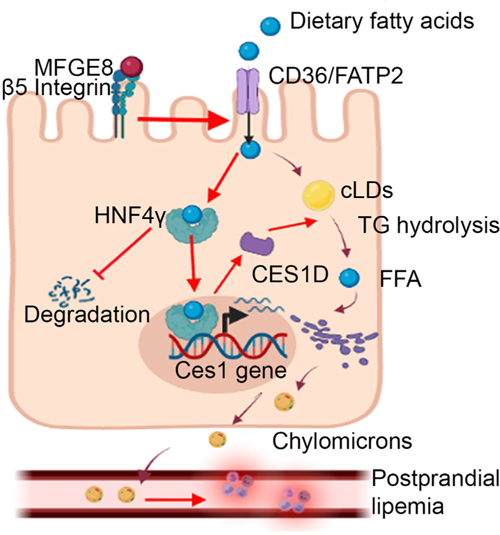

## INTRODUCTION

Intestinal lipid homeostasis has important implications for the development of atherosclerotic heart disease.^1,2^ In addition to absorbing nutrients, the small intestine functions as a lipid storage organ that can limit postprandial serum lipid levels by storing a proportion of absorbed fats in cytoplasmic lipid droplets (cLDs).^3,4^ The clinical relevance of this underappreciated role of the small intestine is evidenced by the stronger correlation of postprandial lipid levels with coronary artery disease as compared with the more commonly measured fasting serum lipid levels.^2^ In obesity, insulin resistance at the level of the intestine removes the suppressive effect of insulin on chylomicron production, resulting in more severe postprandial lipemia.^5^ Humans with visceral obesity also demonstrate more severe post-prandial lipemia and an increased risk of cardiovascular disease.^6^

cLDs are increasingly recognized as dynamic organelles with pleiotropic functions that include prevention of fatty-acid-induced lipotoxicity, serving as platforms for protein binding and degradation and providing a reservoir for hydrophobic molecules important in numerous cellular functions. The small intestine is unique in that enterocytes contain distinct pools of cLDs derived from dietary fat or from lipids taken up from the basolateral circulation.^7,8^ Our current understanding of cLD metabolism is primarily derived from work done in adipocytes and hepatocytes, including the identification of several molecules that associate with and regulate the hydrolysis of triglycerides (TGs) in cLDs. Adipocyte triglyceride lipase (ATGL) is the predominant intracellular hydrolase responsible for cleaving intracellular TG to diacylglycerol^5^ and *Atgl* knockout (KO) mice accumulating TG in multiple tissues.^6^ ATGL and its co-activator CGI-58 are central to a molecular complex including the perilipin family of proteins and G0S2, which orchestrate cLD catabolism in adipocytes and other tissue compartments. Recent work in enterocytes indicates that the ATGL/CGI-58 pathway is active in regulating catabolism of enterocyte cLDs derived from the baso-lateral circulation but not those derived from the diet.^9^ The hydrolase(s) that regulates catabolism of cLDs derived from dietary sources has not been identified.

We recently identified roles for the integrin ligand milk fat globule epidermal growth factor-like 8 (MFGE8) and its receptor, the αvβ5 integrin, in intestinal lipid homeostasis. The MFGE8/integrin pathway links absorption of dietary fats with catabolism of small intestinal cLDs by promoting enterocyte uptake of diet-derived luminal fats^10^ and increasing the activity of enterocyte TG hydrolases, resulting in TG mobilization from cLDs for chylo-micron production.^11^ Interestingly, unlike in the intestine, TG hydrolase activity is unaffected in white adipose tissue or liver of *Mfge8* KO mice,^11^ suggesting that enterocyte-specific pathways regulate catabolism of diet-derived cLDs. In this work we investigated the molecular pathway through which MFGE8 regulates the catabolism of enterocyte cLDs. We identify CES1D, a member of the Ces family of lipases, as the key hydrolase that functions downstream of the MFGE8-integrin complex to mobilize fatty acids from cLD TG stores and regulate chylomicron production. We further show that dietary oleic acid increases expression and activity of CES enzymes through stabilizing protein levels of the transcription factor HNF4γ. The findings provide significant insight into intestinal regulation of postprandial lipid levels.

## RESULTS

### MFGE8 regulates the expression and activity of CES hydrolases

We have previously published that MFGE8 increases enterocyte TG hydrolase activity.^11^ To determine whether this effect is mediated through ATGL, we isolated proximal small intestinal enterocytes from *Atgl* KO mice and assessed the effect of recombinant MFGE8 (rMFGE8) on TG hydrolase activity. rMFGE8 significantly increased TG hydrolase activity in *Atgl* KO enterocytes, and the effect size was similar to that of rMFGE8 on wild-type (WT) enterocyte TG hydrolase activity (Figure S1) and *Mfge8* KO enterocyte TG activity.^11^ We interpret these data to indicate that the effect of MFGE8 on enterocyte TG hydrolase activity does not require ATGL.

**Figure 1.**
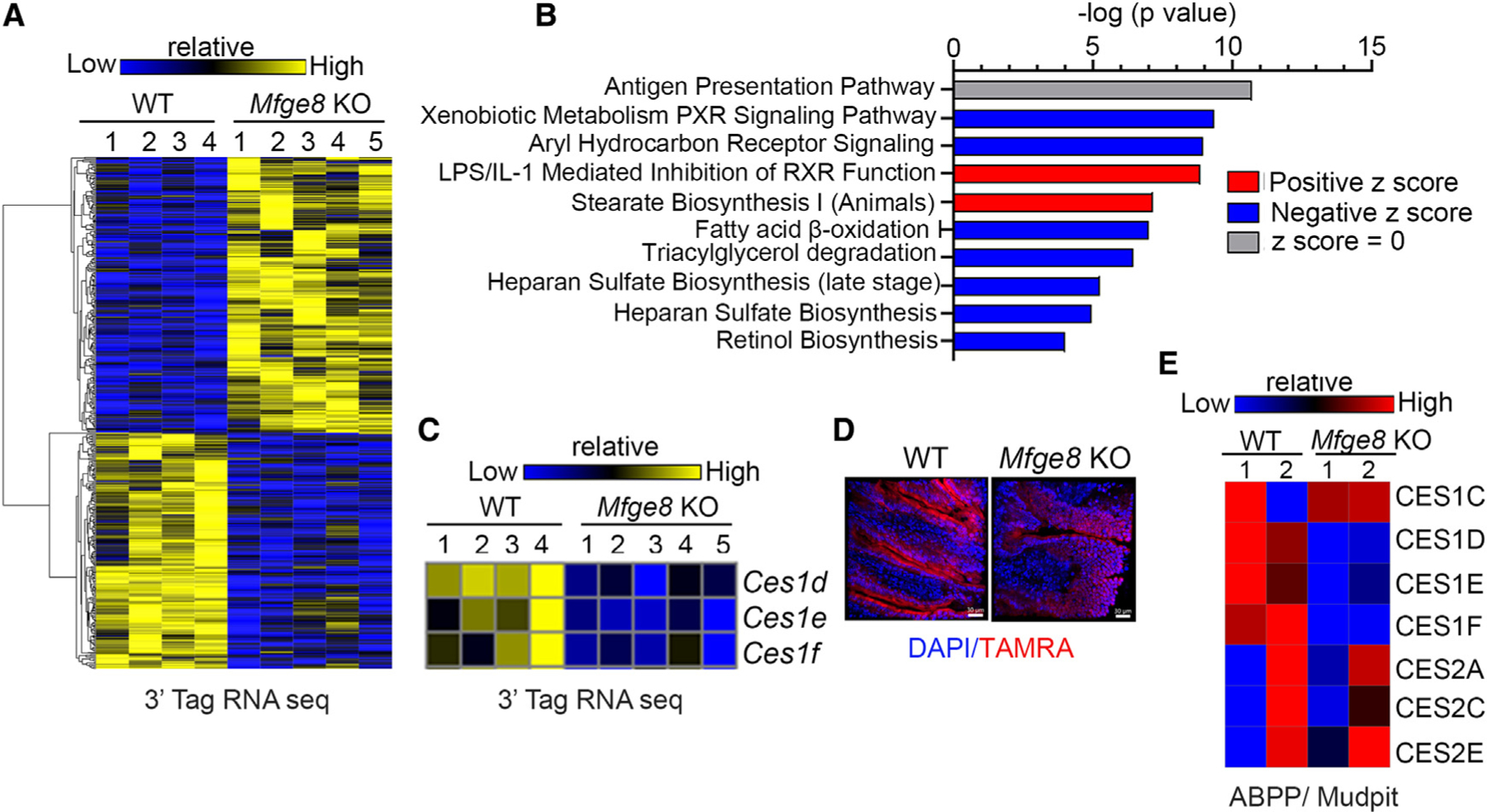
MFGE8 regulates the expression and activity of CES proteins (A–C) 3′ Tag RNA sequencing of WT and *Mfge8* KO mouse primary enterocytes. (A) Heatmap of differentially expressed genes. (B) Ingenuity Pathway Analysis (IPA) of differentially expressed genes showing enriched biological processes. (C) Heatmap showing altered expression of the Ces1 family genes. N = 4 WT and N = 5 *Mfge8* KO 7- to 8-week-old male mice. (D) Confocal imaging of active serine hydrolases in small intestinal cryosections identified with a TAMRA-FP probe (red fluorescence) and counterstained with DAPI (blue). Representative image from two independent experiments. Scale bars, 30 μm. (E) Serine hydrolase ABPP analysis showing differential activities of CES enzymes in WT and *Mfge8* KO primary enterocytes obtained from 8- to 9-week-old male mice. Each sample represents pooled enterocytes from five mice with liquid chromatography-mass spectrometry performed in technical duplicates.

We next took an unbiased approach to investigate which enterocyte TG hydrolases are regulated by MFGE8. We performed 3′ tag RNA sequencing (RNA-seq) of proximal small intestinal enterocytes isolated from WT and *Mfge8* KO mice and identified 530 differentially regulated genes (Figure 1A, accession number GEO: GSE200320). Ingenuity Pathway Analysis (IPA) of these genes showed enrichment for triacylglycerol degradation related signaling (Figure 1B). Interestingly, we observed downregulation of several genes coding for hydrolases belonging to the CES1 family of enzymes in *Mfge8* KO mice (Figure 1C). We next performed activity-based staining^12^ in WT and *Mfge8* KO intestinal cryosections using a fluorescently labeled fluorophosphonate probe (TAMRA-FP) that binds the active confirmation of serine hydrolases. Cryosections from *Mfge8* KO mice showed markedly reduced fluorescence compared with WT controls (Figure 1D) consistent with lower TG hydrolase activity.

To further investigate which CES hydrolases had decreased activity in *Mfge8* KO enterocytes, we performed activity-based protein profiling (ABPP) with a serine hydrolase-specific fluorophosphonate biotin probe (FP-biotin).^13,14^ Consistent with our sequencing data, we found decreased activity for a subset of CES1 enzymes in *Mfge8* KO samples (Figure 1E, accession number MassIVE: MSV000089304). We interpret these data to indicate that MFGE8 regulates intestinal TG hydrolase activity through expression of the CES family of enzymes.

### **MFGE8 regulates the expression of CES hydrolases through the transcription factor HNF4**γ

**Figure 2.**
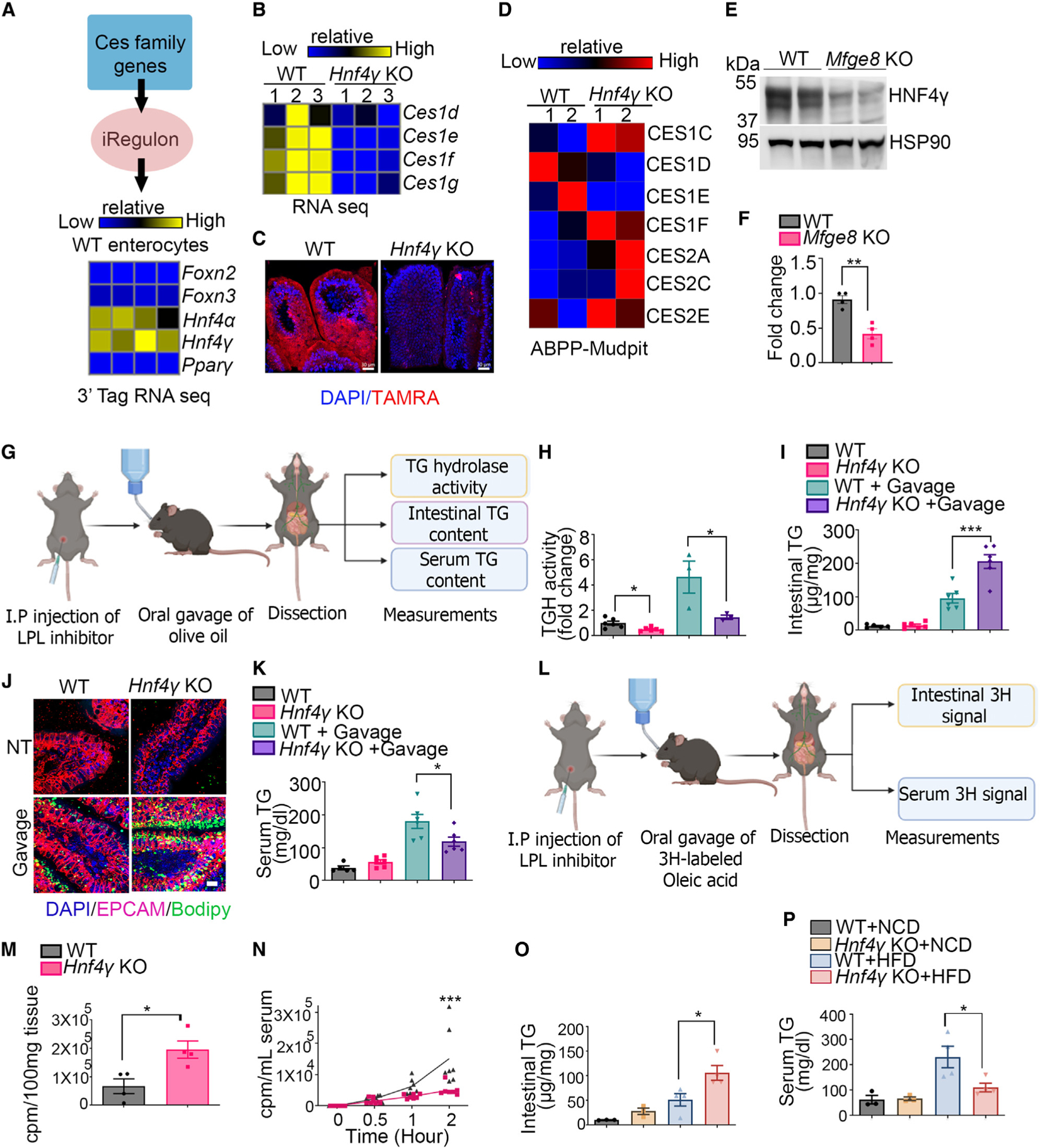
MFGE8 regulates the expression and activity of CES hydrolases through HNF4γ to modulate catabolism of enterocyte cLDs (A) Heatmap of expression of candidate transcription factors identified through the iRegulon database in 3′ tag RNA-seq data of WT enterocytes. (B) Analysis of previously published RNA-seq data (accession number GEO: GSE200320) from WT and *Hnf4γ* KO enterocytes showing differential expression of the Ces1 genes. (C) Confocal imaging of active serine hydrolases identified with TAMRA-FP probe (red fluorescence) and counterstained with DAPI (blue) in WT and *Mfge8* KO intestinal cross-sections. Nuclei were stained with DAPI (blue). Representative image from two independent experiments. Scale bars, 30 μm. (D) Serine hydrolase ABPP analysis showing differential activities of CES enzymes between WT and *Hnf4γ* KO primary enterocytes. N = 2 independent experiments with each sample representing pooled enterocytes from five mice (total of ten mice per group). A mix of 9- to 10-week-old male and female mice were used for this experiment. (E and F) (E) Representative western blot of HNF4γ protein levels in WT and *Mfge8* KO enterocytes from 8- to 10-week-old male and female mice. Experiments were performed two independent times with a total of four mice in each genotype. (F) Densitometric analysis of the western blots (including E). (G) Schema of the experimental design for (H) to (K). (H–K) (H) TG hydrolase (TGH) activity, (I and J) proximal jejunal TG content, and (K) serum TG content at baseline and 2 h after olive oil gavage in primary enterocytes from WT and *Hnf4γ* KO mice (N = 3–6 for H, I, and K). (J) Intestinal tissue sections were stained with Bodipy (green) and anti-Epcam antibody (magenta, N = 2 in each group). Results are from two independent experiments. Scale bar, 30 μm. (L) Schema of the experimental design for (M) and (N). (M and N) (M) ^3^H signal in the proximal jejunum 2 h after oral administration of [^3^H]oleic acid in WT and *Hnf4γ* KO mice. (N) ^3^H signal in the serum over time after oral administration of [^3^H]oleic acid. N = 4–7 mice per group from two independent experiments. A mix of 7- to 10-week-old male and female mice were used for these experiments. (O and P) (O) Intestinal and (P) serum TG content of 8-week-old WT and *Hnf4γ* KO mice after 3 weeks on an HFD or normal chow diet and following a 12-h fast. N = 3–4, data are expressed as mean ± SEM; *p < 0.05, **p < 0.01, ***p < 0.001. Data in (H), (I), (K), (O), and (P) were analyzed by one-way ANOVA followed by Bonferroni’s post test. Data in (F) and (M) were analyzed by unpaired Student’s t test. Data in (N) were analyzed by two-way ANOVA followed by Bonferroni’s post test.

We next utilized the iRegulon database to identify putative candidate transcription factors that could mediate the effect of MFGE8 on *Ces* gene expression and cross-referenced these with transcription factors expressed in WT enterocytes from our 3′ tag RNA-seq data. From this analysis, we found highest expression of the HNF4 family of transcription factors (consisting of HNF4α and HNF4γ) in WT enterocytes (Figure 2A). We subsequently analyzed available RNA-seq data from a recent publication comparing gene expression of WT, *Hnf4γ* KO, and *Hnf4α* KO murine enterocytes.^15^ We found altered expression of multiple *Ces1* family genes in *Hnf4γ* KO (Figure 2B) but not in *Hnf4α* KO enterocytes (Figure S2).

Next, we performed activity-based staining of serine hydro-lases in WT and *Hnf4γ* KO proximal small intestinal cryosections and found a marked reduction in the hydrolase signal in the *Hnf4γ* KO group (Figure 2C). We also performed ABPP with FP-biotin and found that loss of HNF4γ led to reduced enzymatic activity of multiple CES1 subfamilies (Figure 2D, accession number MassIVE: MSV000089304) including CES1D. We next studied whether MFGE8 regulates HNF4γ transcript or protein expression. HNF4γ transcript was unchanged in *Mfge8* KO and WT proximal small intestinal enterocytes in our 3′ tag RNA-seq dataset (GEO: GSE200320). However, there was a marked reduction in HNF4γ protein levels in *Mfge8* KO enterocytes (Figures 2E and 2F). We interpret these data to indicate that MFGE8 modulates CES enzyme gene transcription by regulating HNF4γ protein levels.

### HNF4γ regulates catabolism of enterocyte cLDs

To investigate the functional role of HNF4γ in enterocyte cLD homeostasis, we challenged WT and *Hnf4γ* KO mice with olive oil gavage (Figure 2G) and evaluated proximal small intestinal enter-ocyte TG hydrolase activity, proximal small intestinal tissue TG content, and serum TG levels. *Hnf4γ* KO enterocytes had significantly reduced TG hydrolase activity at baseline and 2 h after olive oil gavage (Figure 2H). Two hours after gavage, the increase in hydrolase activity was coupled with greater proximal small intestinal tissue TG content and lower serum TG levels (Figures 2I–2K). We next administered ^3^H-labeled oleic acid by gavage to WT and *Hnf4γ* KO mice in the presence of the lipoprotein inhibitor tyloxapol (to prevent catabolism of serum TG) and measured the radioactive signal in the proximal small intestinal tissue and in the serum 2 h later (Figure 2L). *Hnf4γ* KO mice had greater proximal small intestinal radioisotope accumulation and reduced serum radiolabel (Figures 2M and 2N). The *Hnf4γ* KO mice were then fed a high-fat diet (HFD) or a control diet for 3 weeks. After a 12-h fast, the *Hnf4γ* KO mice fed an HFD had greater proximal small intestinal tissue TG content and lower serum TG content as compared with WT mice on a normal chow diet (Figures 2O and 2P). Of note, *Hnf4γ* KO mice exposed to acute or chronic fat challenges phenocopied our previous findings with *Mfge8* KO mice,^11^ supporting the role of HNF4γ in catabolism of intestinal cLDs.

### CES1D regulates hydrolysis of enterocyte cLDs

**Figure 3.**
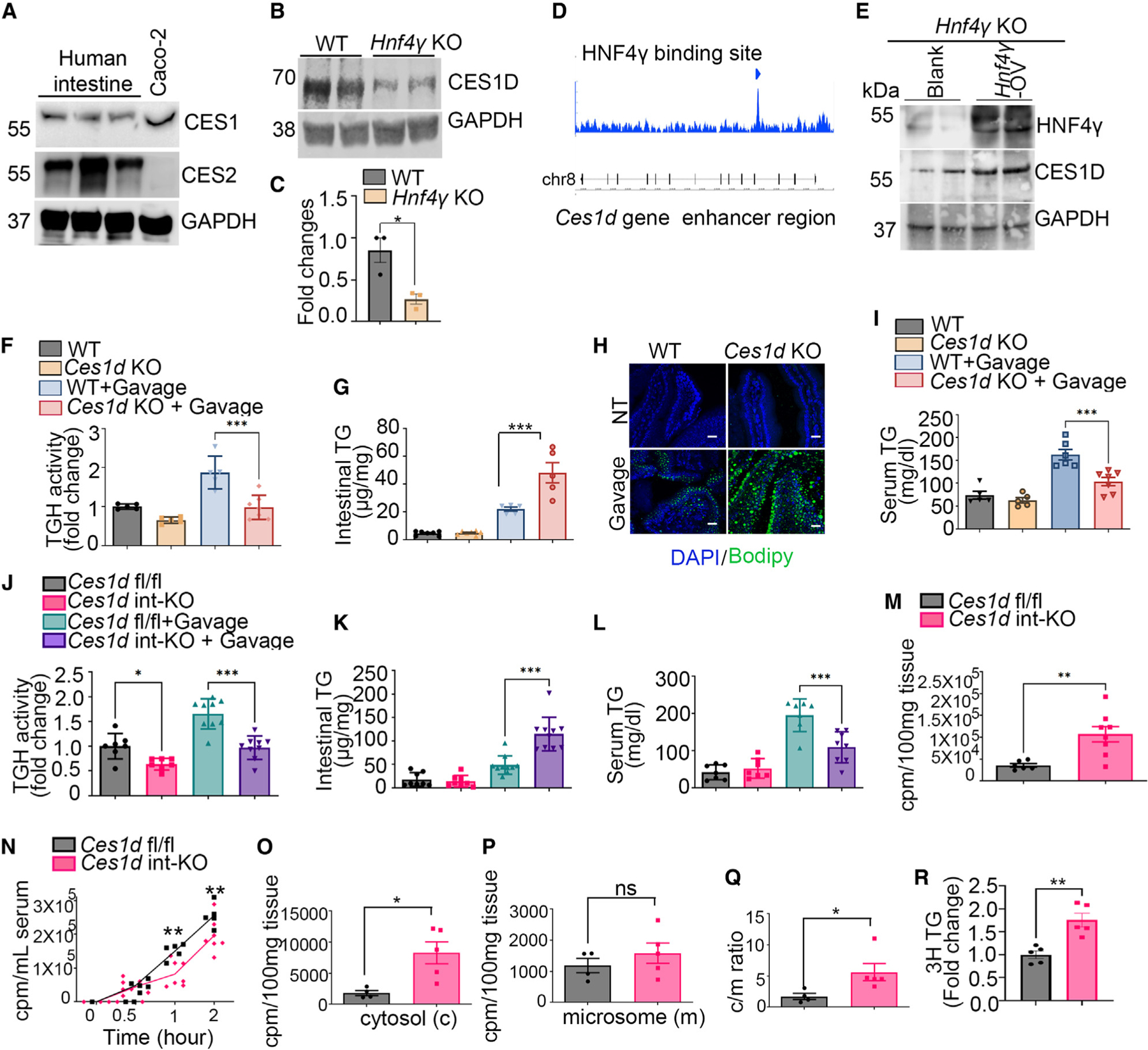
CES1D regulates hydrolysis of enterocyte cLDs (A) Western blot of CES1 and CES2 in enterocyte lysates from human patients with inflammatory bowel disease. Caco-2 lysates serve as a positive control for CES1 expression with GAPDH a loading control. N = 3 independent patient samples. (B) Representative western blot of CES1D in WT and *Hnf4γ* KO enterocyte lysates from two independent experiments, N = 3 mice in total. (C) Densitometric analysis of the western blots of CES1D (including B). (D) Analysis of previously published ChIP-seq data showing binding sites for HNF4γ on the promoter/enhancer regions of Ces family genes (location: chr8: 93, 892, 883–893, 916, 999). (E) Western blot showing HNF4γ and CES1D protein level in *Hnf4γ* KO mice intestine segments incubated with *Hnf4γ*-expressing or control adenovirus (*Hnf4γ*-AV or Blank). Western blot is representative of three independent experiments with N = 4 *Hnf4γ* KO mice in total per experimental group. (F, G, and I) (F) Proximal small intestinal enterocyte TG hydrolase activity, (G) proximal small intestinal tissue TG content, and (I) serum TG content at baseline and 2 h after olive oil gavage in WT and *Ces1d* KO mice. N = 5–7 mice in each group. Results are from three independent experiments. (H) Proximal small intestinal tissue sections from the same group of mice (F, G, and I) were stained with DAPI (blue) and Bodipy (green). Scale bars, 30 μm. (J–L) (J) TG hydrolase activity in primary enterocytes, (K) TG content in the proximal jejunum, and (L) TG content in the serum at baseline and 2 h after olive oil gavage in WT and *Ces1d* int-KO mice, N = 7–10 mice in each group. Data merged from four independent experiments. (M) ^3^H signal in the proximal small intestinal tissue 2 h after oral administration of [^3^H]oleic acid. Results are from two independent experiments, N = 6–8 mice in each group. (N) ^3^H signal in the serum after oral administration of [^3^H]oleic acid over time in control and *Ces1d* int-KO mice. Results are from three independent experiments, N = 5–7 mice in each group. (O–R) ^3^H signal in the (O) cytosolic fraction and (P) microsomal fraction, and (Q) the ratio of cytosolic to microsomal radioactive signal in proximal small intestinal tissue and (R) ^3^H signal in the proximal small intestinal TGs separated by TLC from control and *Ces1d* int-KO mice 2 h after oral gavage of ^3^H-labeled oleic acid. N = 4–5 mice in each group for (O) and (P) and N = 5 mice in each group for (R). Data in (F), (G), and (I) to (L) were analyzed by one-way ANOVA followed by Bonferroni’s post test. Data in (C), (M), and (O) to (R) were analyzed by unpaired Student’s t test. Data in (N) were analyzed by two-way ANOVA followed by Bonferroni’s post test. All data are expressed as mean ± SEM. *p < 0.05, **p < 0.01, ***p < 0.001.

We were next interested in understanding whether reduced expression of CES enzymes leads to impaired TG hydrolase activity in *Mfge8* KO and *Hnf4γ* KO enterocytes. The human genome contains six CES genes (*CES1*, *CES2*, *CES3*, *CES4A*, *CES5A*, and *CES1P1*). The mouse genome contains a larger number of CES proteins (20 have been annotated) due to tandem gene duplication.^16^ Of these, CES1d, CES1f, CES1g, CES2a, CES2b, CES2c, and CES2e have known TG hydrolase activity.^16–18^ Expression of CES2 in the human intestine is well documented.^19,20^ To delineate whether CES1 protein is expressed in human intestine, we performed western blot of human small bowel epithelial cell lysates prepared from intestinal resections of patients with inflammatory bowel disease. Both CES1 and CES2 were expressed in these lysates (Figure 3A). Caco-2 cell lysates, a human colon carcinoma cell line known to express high levels of CES1 and low levels of CES2,^21^ served as a positive control for these western blots. We next demonstrated that small interfering RNA-mediated knockdown of Ces1 gene expression in differentiated Caco-2 cells reduces TG hydrolase activity at baseline, indicating that CES1 regulates TG hydrolase activity and corroborating the previously published literature^22,23^ (Figure S3A). We validated successful knockdown of CES1 by western blot using anti-CES1 antibody (Figure S3B).

We next focused on Ces1D, since its expression (Figures 1C and 2B) and activity (Figures 1E and 2D) were significantly decreased in *Mfge8* and *Hnf4γ* KO enterocytes and because it is the closest murine ortholog of human CES1.^16^ Furthermore, Ces1D functions as a TG hydrolase,^24,25^ and a recent report showed high activity of this enzyme in the proximal small intestine.^26^ CES1D protein levels by western blot were markedly reduced in *Hnf4γ* KO enterocytes (Figures 3B and 3C). We next analyzed data from recently published work looking at transcriptional targets of HNF4γ utilizing chromatin immunoprecipitation sequencing (ChIP-seq) in mouse enterocytes^16^ and identified transcriptional binding sites for HNF4γ in the enhancer regions of *Ces1d* (Figures 3D and S4). We next used an adenoviral vector to express exogenous HNF4γ for 24 h in Hnf4γ KO intestines *ex vivo* and subsequently probed by western blot for HNF4γ and CES1D protein expression. Forced expression of HNF4γ in *Hnf4*γ KO enterocytes rescued CES1D protein levels (Figure 3E).

We next evaluated enterocyte LD homeostasis in *Ces1d* KO mice. Global *Ces1d* KO mice had reduced proximal small intestinal enterocyte TG hydrolase activity at baseline and after olive oil gavage (Figure 3F), coupled with increased proximal small intestinal tissue TG content (Figures 3G and 3H) and reduced serum TG levels (Figure 3I). Enterocyte-specific deletion of *Ces1d* (*Ces1d* int-KO using villin-Cre transgene) had significantly reduced proximal small intestinal enterocyte TG hydrolase activity at baseline and after olive oil gavage (Figure 3J), which was associated with increased proximal small intestinal tissue TG content and reduced serum TG levels 2 h after olive oil gavage (Figures 3K and 3L). Oral gavage of ^3^H-labeled oleic acid to *Ces1d* int-KO mice increased proximal small intestinal tissue radioactivity (Figure 3M) and reduced it in serum as compared with controls (Figure 3N). Together, these data indicate that mice with global or intestine-specific *Ces1d* deletion phenocopy *Mfge8* KO^11^ and *Hnf4γ* KO mice (Figure 2) in their response to the impact of olive oil gavage on intestinal and serum lipids.

After olive oil gavage, Mfge8 KO mice accumulate TG in the cytosolic as opposed to microsomal fraction of enterocytes, consistent with altered cLD homeostasis rather than impaired chylomicron assembly and/or secretion.^11^ We therefore evaluated the intracellular location of accumulated TG in *Ces1d* int-KO mice 2 h after ^3^H-labeled oleic acidgavagebyfractionating jejunal enter-ocytes into cytosolic and microsomal components and measuring the radiolabel signal in each fraction. As with *Mfge8* KO mice,^11^ *Ces1d* int-KO miceaccumulated radiolabelin the cytosolic fraction in comparison with WT controls and with no apparent differences in the microsomal fraction, indicating impaired cLD homeostasis in *Ces1d* int-KO mice (Figures 3O–3Q). We confirmed the relative enrichment of cytosolic and microsomal fractions by western blotting for the cytosolic protein GAPDH and microsomal protein BIP (Figure S5). We also measured the incorporation of the ^3^H radio-label into TGs in proximal small intestinal tissues. We extracted lipids from the control and *Ces1d* int-KO mouse proximal small intestinal tissue 2 h after ^3^H-labeled oil gavage, performed thin-layer chromatography (TLC) to separate TGs on a silica gel, and measured the ^3^H intensity in the TG fraction. We observed enhanced incorporation of the ^3^H radiolabel in TG extracted from *Ces1d* int-KO mice compared with control mice (Figure 3R). Taken together, these data indicate that CES1D regulates the hydrolysis of diet-derived cLDs.

### MFGE8 regulates TG hydrolase activity through CES1D

**Figure 4.**
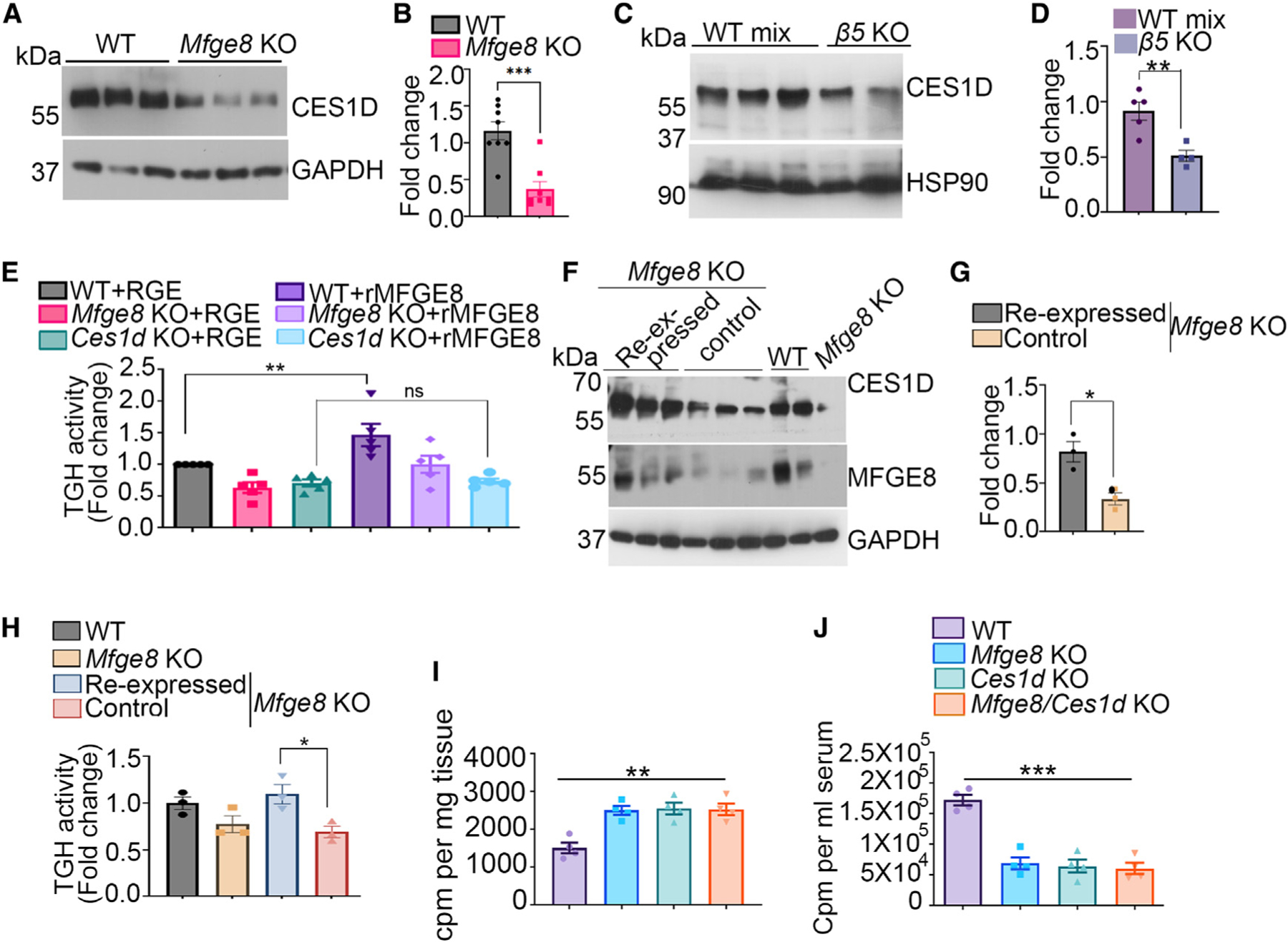
MFGE8 regulates TG hydrolase activity through CES1D (A and B) (A) Representative western blot showing CES1D protein level in WT and *Mfge8* KO primary enterocytes from three independent experiments. GAPDH was used as loading control. N = 8 mice per group. (B) Densitometric analysis of the western blots (including A). (C) Representative western blot showing CES1D protein level in WT and *β5* KO primary enterocytes from two independent experiments. HSP90 was used as loading control. N = 5 WT and N = 4 *β5* KO mice in total. (D) Densitometric analysis of the western blots of CES1D protein (including C). (E) TG hydrolase activity in WT, *Ces1d* KO, and *Mfge8* KO primary enterocytes 1 h after incubation with rMFGE8 or RGE. N = 5 independent experiments. (F) Western blot of CES1D and MFGE8 protein levels in proximal small intestinal enterocytes of *Mfge8* KO mice with transgenic inducible expression of MFGE8 in enterocytes (MFGE8 re-expressed, Vil rtTA^+^ TetO Mfge8^+^) and single transgenic (control), WT, and *Mfge8* KO enterocyte controls. GAPDH was used as loading control. (G) Densitometric analysis of the blot presented in (F). (H) TG hydrolase activity in primary enterocytes isolated from the same groups of mice in (E) and (F). N = 3 mice in each group. (I and J) ^3^H signal in (I) the proximal jejunum and (J) serum 2 h after oral administration of [^3^H]oleic acid to WT, *Mfge8* KO, *Ces1d* KO, and *Mfge8/Ces1d* double-KO mice. N = 3–4 mice in each group. All data are expressed as mean ± SEM. *p < 0.05, **p < 0.01, ***p < 0.001. Data in (B), (D), and (G) were analyzed by unpaired t test. Data in (E) and (H) to (J) were analyzed by one-way ANOVA followed by Bonferroni’s post test.

To determine whether MFGE8 and the αvβ5 integrin modulate CES1D protein levels, we performed western blot in *Mfge8* KO and *β5* KO enterocytes and found marked reduction of CES1D in both populations (Figures 4A–4D). To directly assess whether the effect of MFGE8 on cLD catabolism is mediated through CES1D, we evaluated the ability of rMFGE8 to increase TG hydrolase activity in *Ces1d* KO enterocytes. While rMFGE8 significantly increased TG hydrolase activity in WT and *Mfge8* KO enterocytes, it had no effect on *Ces1d* KO enterocytes (Figure 4E). We used a mutated Mfge8 protein construct (RGE) that cannot bind integrins^10^ as a negative control (Figure 4E). We next assessed whether inducible expression of MFGE8 in enterocytes in *Mfge8* KO mice using a tetracycline-inducible system^27^ modulated CES1D protein levels. Inducible expression of MFGE8 rescued the loss of CES1D protein levels (Figures 4F and 4G) as well as the TG hydrolase activity (Figure 4H). We then assessed cLD catabolism in mice with global deletion of both *Ces1d* and *Mfge8* (*Ces1d/Mfge8* KO). We administered ^3^H-labeled oleic acid by gavage to WT, *Ces1d* KO, *Mfge8* KO, and *Ces1d/Mfge8* KO mice and quantified ^3^H radiolabel in the small intestine and serum 2 h after gavage (Figures 4I and 4J). *Ces1d/Mfge8* KO mice had a similar increase in proximal small intestinal radiolabel and a similar reduction in serum radiolabel as *Mfge8* and *Ces1d* KO mice, indicating that the loss of both alleles had no additive effect (Figures 4I and 4J). Together, these data indicate that MFGE8 modulates proximal small intestinal enterocyte TG hydrolase activity in large part through CES1D.

### **MFGE8 links fatty acid absorption to LD catabolism through HNF4**γ

**Figure 5.**
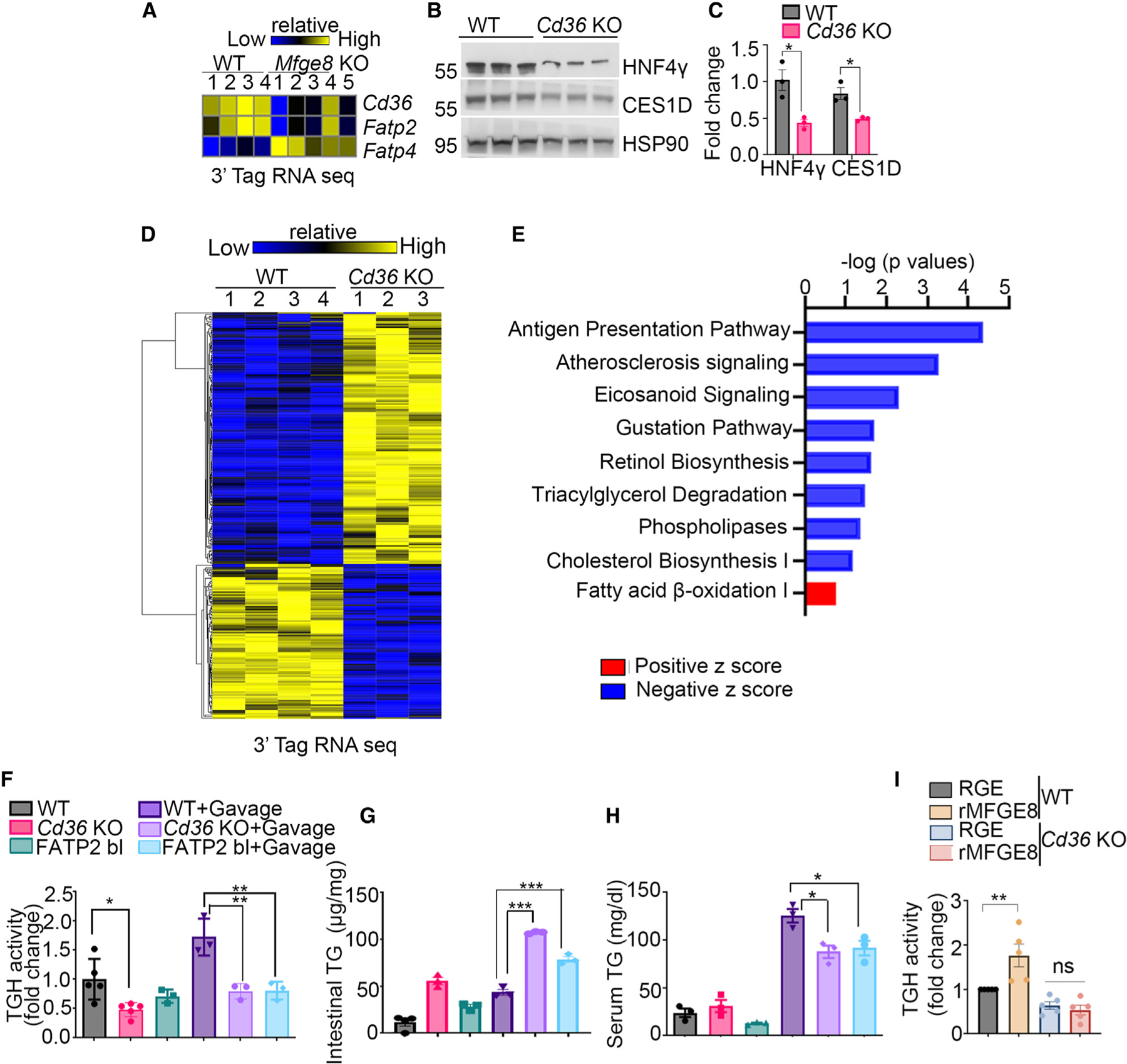
MFGE8 links fatty acid absorption to LD catabolism through HNF4γ (A) Heatmap showing differentially expressed fatty acid transporters in WT and *Mfge8* KO primary enterocytes from 3′ tag RNA sequencing (GEO: GSE200320). (B and C) (B) Western blot of HNF4γ and CES1D protein level in WT and *Cd36* KO primary enterocytes. N = 3 mice in each group. (C) Densitometric analysis of the western blot in (B). (D and E) (D) Heatmap and (E) IPA of differentially expressed genes in 3′ tag RNA-seq of WT and *Cd36* KO proximal small intestinal tissue. (F–H) Primary enterocyte (F) TG hydrolase activity, (G) TG content in the proximal small intestinal tissue, and (H) serum TG content at baseline and 2 h after acute fat challenge of WT, *Cd36* KO, and WT mice treated with pharmacological inhibitor of FATP2 (FATP2 bl). (I) TG hydrolase activity in WT and *Cd36* KO primary enterocytes 1 h after incubation with rMFGE8 or RGE. N = 5 independent experiments. Data are expressed as mean ± SEM. *p < 0.05, **p < 0.01, ***p < 0.001. Data in (C) were analyzed by unpaired t test. Data in (F) to (I) were analyzed by one-way ANOVA followed by Bonferroni’s post test.

We have previously shown that MFGE8 promotes absorption of dietary fatty acids in the small intestine. HNF4γ is a nuclear hormone receptor that constitutively binds saturated and *cis-* monounsaturated fatty acids of 14–18 carbons.^28^ We therefore examined whether MFGE8-mediated fatty acid absorption impacts the activity of HNF4γ. In our 3′ tag RNA-seq data (GEO: GSE200320), we found decreased expression of *Cd36* and *Fatp2* (Figure 5A), two fatty acid transporters that are active in the small intestine,^29–32^ in *Mfge8* KO enterocytes. We therefore assessed whether genetic deletion of *Cd36* impacts HNF4γ. We observed a marked reduction in HNF4γ protein level in *Cd36* KO proximal small intestinal enterocytes (Figures 5B and 5C). Moreover, genetic deletion of *Cd36* also caused a reduction in CES1D protein level in proximal small intestinal enterocytes (Figures 5B and 5C).

We next performed 3′ tag RNA-seq of WT and *Cd36* KO proximal small intestinal tissue (Figure 5D). IPAs of differentially expressed genes indicated enrichment of TG degradation processes (Figure 5E). Furthermore, TG hydrolase activity was significantly decreased in *Cd36* KO proximal small intestinal enterocytes at baseline and after acute fat challenge (Figure 5F). Pharmacological blockade of FATP2 in WT mice also suppressed proximal small intestinal enterocyte TG hydrolase activity after acute fat challenge (Figure 5F). Both *Cd36* KO mice and WT mice treated with a pharmacological inhibitor of FATP2 accumulated lipids in the proximal small intestinal tissue and had lower serum TG level after an acute fat challenge as compared with WT controls (Figures 5G and 5H). To further assess whether the effect of MFGE8 on LD catabolism involves CD36, we evaluated the ability of rMFGE8 to increase TG hydro-lase activity in *Cd36 KO* enterocytes. While rMFGE8 significantly increased TG hydrolase activity in WT enterocytes, it had no effect on Cd36 KO enterocytes (Figure 5I). These data suggest that the effects of MFGE8 on enterocyte HNF4γ protein levels and LD catabolism are linked through MFGE8/CD36-dependent fatty acid absorption.

### Fatty acid stabilizes HNF4γ protein to activate transcription of *Ces* genes

**Figure 6.**
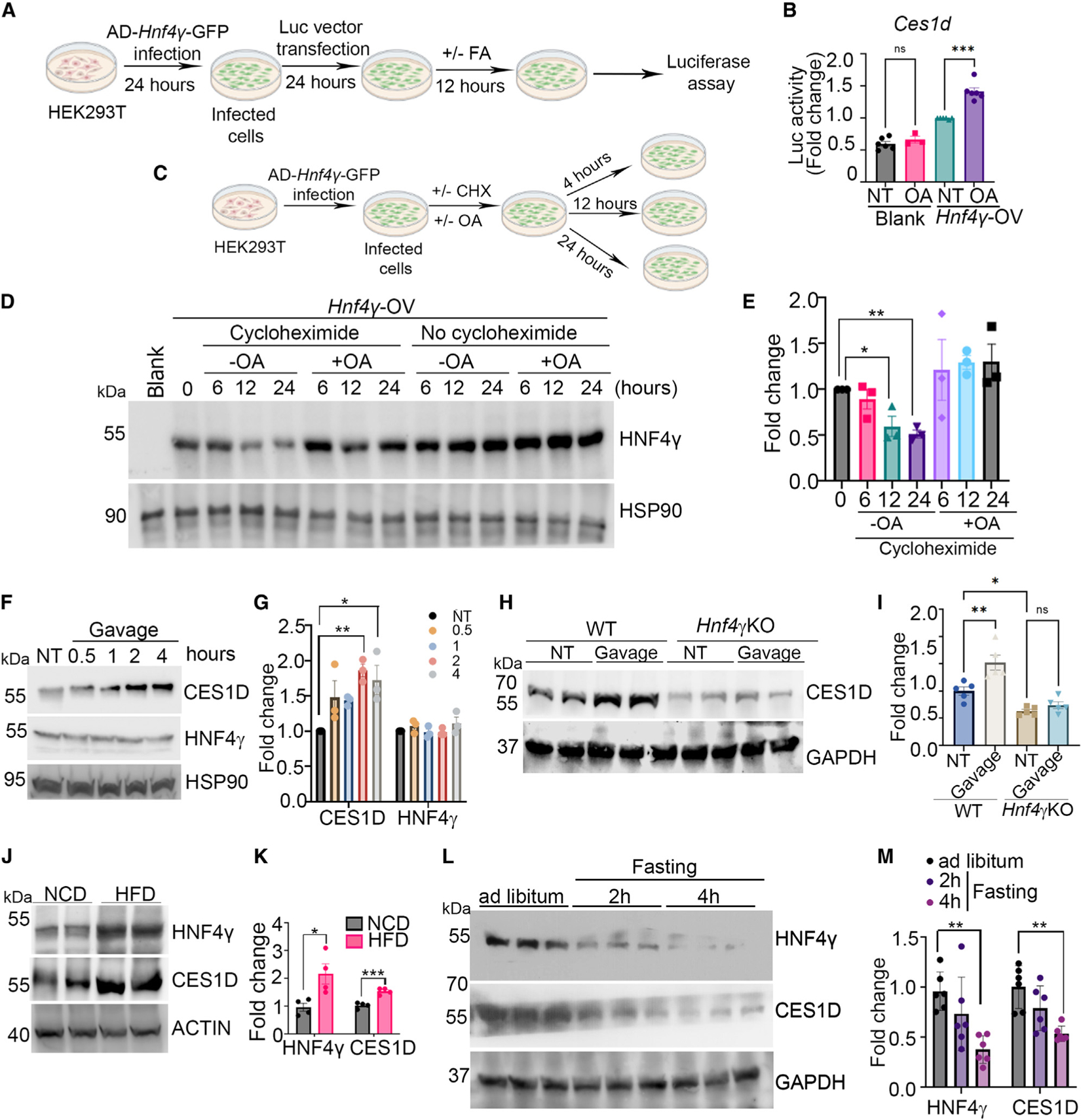
Fatty acid stabilizes HNF4γ protein to activate transcription of Ces genes (A) Schematic representation of the method used for dual luciferase assay presented in (B). (B) Data showing normalized luciferase activity of the Ces1d gene enhancer in the presence of oleic acid in HEK293 cells with adenoviral overexpression of *Hnf4γ* (*Hnf4γ*-OV). Cells infected with a blank adenovirus (Blank) were used as a negative control. N = 3–6 independent experiments. (C) Schematic representation of the method used for the cycloheximide chase assay presented in (D). (D) Representative western blot showing HNF4γ protein levels 6, 12, and 24 h after treatment with oleic acid or DMSO control in the presence and absence of cycloheximide in HEK293 cells. Western blot is representative of three independent experiments. (E) Densitometric analysis of HNF4γ protein levels (including D). (F) Representative western blot showing CES1D and HNF4γ protein levels at baseline and 30 min, 1 h, 2 h, and 4 h after oral gavage of olive oil. Western blot is representative of three independent experiments. (G) Densitometric analysis of CES1D and HNF4γ protein levels (including F). N = 1 mice per group per experiment (total of three mice in each group). (H) Representative western blot showing CES1D protein level in WT and *Hnf4γ*KO intestine at baseline and 2 h after olive oil gavage. Data represent two independent experiments. (I) Densitometric analysis of CES1D protein levels (including H). N = 2–3 mice per group per experiment (total of five mice in each group). (J) Representative western blot showing HNF4γ and CES1D protein level in the small intestine of mice on a normal chow or high-fat chow diet. Results are from two independent experiments. (K) Densitometric analysis of the western blots of HNF4γ and CES1D (including J). N = 2 mice per group per experiment (total of four mice in each group). (L) Representative western blot showing HNF4γ and CES1D protein level in the small intestine of mice on a normal chow diet and 2 h and 4 h after fasting. Data represent two independent experiments. (M) Densitometric analysis of the western blots of HNF4γ and CES1D (including L). N = 3 mice per group per experiment (total of six mice in each group). A mix of 5- to 7-week-old male and female mice were used for these experiments. All data are expressed as mean ± SEM. *p < 0.05, **p < 0.01, ***p < 0.001. Data in (K) were analyzed by unpaired t test. Data in (B), (E), (G), (I), and (M) were analyzed by one-way ANOVA followed by Bonferroni’s post test.

We next assessed whether oleic acid activates HNF4γ-mediated transcription of *Ces* genes associated with lipid catabolism. We cloned the 500-bp region of the putative enhancer regions of *Ces1d* into a luciferase vector and performed a dual luciferase activity assay in control and HEK293 cells overexpressing HNF4γ (via adenovirus) in the presence and absence of oleic acid (Figure 6A). Cells with HNF4γ overexpression had significantly increased luciferase activity for *Ces1d*. Interestingly, oleic acid further induced transcription of *Ces1d* (Figure 6B). We next performed a 24-h cycloheximide pulse-chase experiment in HEK293 cells in which we overexpressed HNF4γ by adenovirus and subsequently incubated cells with oleic acid (Figure 6C). HNF4γ protein levels decreased at the 12-h time point in the presence of cycloheximide, but addition of oleic acid prevented this decay (Figures 6D and 6E). We interpret these data to indicate that oleic acid induces *Ces1d* transcription by stabilizing enterocyte HNF4γ protein levels.

To evaluate physiological regulation of the HNF4γ-CES1D axis, we assessed protein levels of CES1D and HNF4γ in the total proximal small intestinal tissue lysates 30 min, 1 h, 2 h, and 4 h after olive oil gavage. CES1D protein levels increased 2 h after olive oil gavage with no notable change in HNF4γ protein levels (Figures 6F and 6G). We next determined whether CES1D induction is HNF4γ dependent by quantifying CES1D protein level 2 h after olive oil gavage in WT and Hnf4γ KO mice. WT, but not Hnf4γ KO mice, had an increase in CES1D protein after olive oil gavage, indicating that this effect is HNF4γ dependent (Figures 6H and 6I). We next determined how chronic intake of an HFD impacts HNF4γ and CES1D expression. After 3 weeks on an HFD, proximal small intestinal protein levels of HNF4γ and CES1D were increased in comparison with mice fed normal chow (Figures 6J and 6K). We next determined whether HNF4γ and CES1D protein levels in the proximal small intestinal tissue were affected by fasting after fasting mice for different time periods. Fasting mice for 4 h reduced HNF4γ and CES1D protein levels, indicating that absence of dietary fatty acid suppresses the pathway (Figures 6L and 6M). Taken together, we interpret these data to indicate that stabilization of HNF4γ protein levels by dietary fatty acids drives the increase in CES1D.

### The effect of MFGE8 of cLD catabolism is unique to diet-derived cLDs

To determine whether MFGE8 also modulates cLDs derived from the basolateral circulation, we administered [^3^H]oleic acid intraperitoneally to *Mfge8* KO and WT mice and quantified the radioactive signal in the small intestine. Interestingly, we did not observe differences when comparing *Mfge8* KO and WT samples (Figure S6A). We also performed cell fractionation and measured the radioactive signal in cytosolic and microsomal fractions and found no significant differences between *Mfge8* KO and WT samples. (Figures S6B–S6D). Additionally, we extracted proximal small intestinal lipids, performed TLC to separate TGs, and measured the intensity of the radiolabel in TGs. WT and *Mfge8* KO mice did not show any significant difference in the ^3^H-labeled proximal small intestinal TGs (Figure S6E). These data support the interpretation that the effect of MFGE8 on cLD hydrolysis is restricted to diet-derived cLDs.

### β**5 blockade reduces the extent of postprandial lipemia**

**Figure 7.**
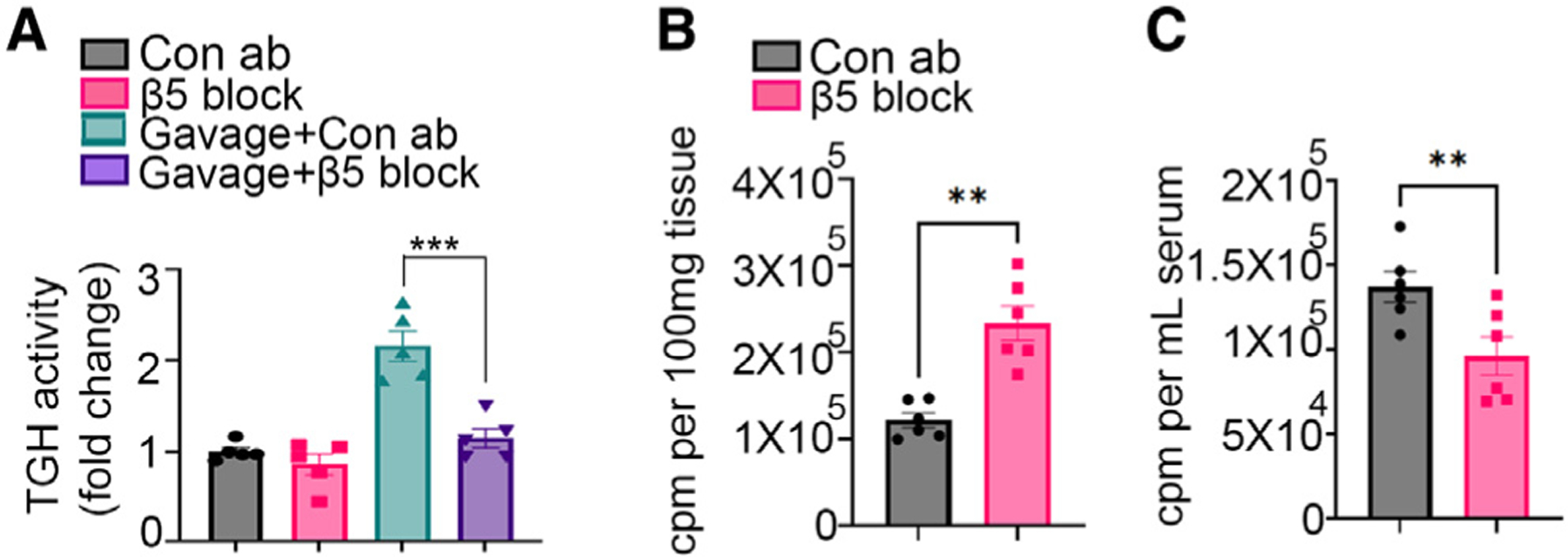
β5 blockade impairs hydrolysis of enterocyte cLDs (A) TG hydrolase activity in primary enterocytes at baseline and 2 h after acute fat challenge inWT mice treated with either β5 blocking antibody or control antibody. N = 5 mice in each group. Results are from two independent experiments. (B and C) ^3^H signal in the (B) proximal small intestinal tissue and (C) serum 2 h after oral administration of [^3^H]oleic acid in WT mice treated with either β5 blocking antibody or control antibody. N = 6 mice in each group. Data merged from two independent experiments. A mix of 5- to 7-week-old male and female mice were used for these experiments. Data are expressed as mean ± SEM. **p < 0.01, ***p < 0.001. Data in (A) were analyzed by one-way ANOVA followed by Bonferroni’s post test. Data in (B) and (C) were analyzed by unpaired t test.

Finally, we determined how systemic blockade of β5 impacts postprandial lipemia in WT mice. Intraperitoneal injection of β5 blocking antibody to WT mice reduced TG hydrolase activity in the proximal small intestinal enterocytes after an acute olive oil gavage as compared with isotype control antibody (Figure 7A). Next, we administered ^3^H-labeled oleic acid by gavage to WT mice treated with either β5 blocking or control antibody in the presence of the lipoprotein inhibitor tyloxapol and subsequently measured proximal small intestinal and serum levels of the radio-label 2 h after gavage. β5 blockade significantly increased the ^3^H signal in the proximal small intestine tissue and decreased the ^3^H signal in serum compared with control mice (Figures 7B and 7C). Taken together, these data indicate that β5 blockade reduces the extent of postprandial lipemia by preventing hydrolysis of proximal small intestinal enterocyte cLDs.

## DISCUSSION

Enterocytes are unique polarized cells that absorb fatty acids from two distinct cellular pools: circulating fatty acids from the basolateral surface and dietary fatty acids from the apical surface.^9^ Absorbed fatty acids can be catabolized through β-oxidation, packaged into chylomicrons for delivery through the circulation to peripheral organs, or retained in the enterocyte as part of cLDs. Storage of fatty acids in cLDs modulates the risk of developing atherosclerotic disease by minimizing the extent of postprandial lipemia, particularly in the setting of a fat-rich diet. The importance of this regulatory mechanism is evident when one considers that humans with obesity and/or diabetes characteristically have exaggerated postprandial lipemia^33^ and a marked increase in the risk of developing coronary artery disease. Of note, oxidation of chylomicron remnants is particularly pro-atherogenic,^34,35^ providing one rationale for the observation that serum lipid levels after a meal have a stronger correlation with coronary artery disease than fasting serum lipid levels.^2^

Enterocytes incorporate fatty acids derived from the circulation or the diet into unique cLD pools with disparate fates.^7,36^ cLDs derived from the circulation are primarily utilized for phospholipid synthesis or β-oxidation while those from the diet are primarily incorporated into TGs used for chylomicron production.^7,36^ Catabolism of each cLD pool occurs through distinct molecular pathways. Hydrolysis of enterocyte cLDs derived from the circulation, but not from dietary lipids, occurs via the same molecular pathways utilized by adipocytes and is centered on the ATGL/CGI-58 complex.^5,6,9,37^

We have previously shown that MFGE8 links the absorption of dietary fats with mobilization of fatty acids from cLDs for chylomicron production through ligation of αv integrins.^10,11^ Our current work demonstrates that the effect of the MFGE8-integrin axis on cLDs is independent of ATGL, since rMFGE8 significantly increases enterocyte TG hydrolase activity in *Atgl* KO enterocytes. Using an unbiased approach, we found differential expression of the CES enzyme family of hydrolases in *Mfge8* KO enterocytes and subsequently showed that one member, CES1D, functions downstream of the MFGE8-integrin axis in mediating cLD hydrolysis. While the human genome contains six CES genes, tandem gene duplication has led to 20 annotated Ces enzymes in the mouse genome.^16^ The CES1 family in mice consists of eight members, three of which had decreased expression in *Mfge8* KO enter-ocytes (*Ces1d, -e,* -*f*). We focused on CES1D because it is the closest murine ortholog of human *CES1* and has high activity in the proximal intestine^26^ where the bulk of chylomicrons are generated. Whether CES1D mediates the entirety of the effect of MFGE8 on enterocyte TG hydrolase activity is difficult to ascertain given the number of CES genes that have altered expression or activity in *Mfge8* KO enterocytes. However, our data indicate that CES1D mediates the bulk of the effects of MFGE8 on enterocyte TG hydrolysis. This conclusion is supported by how closely the *Ces1d* KO mice phenocopy *Mfge8* KO mice in their response to acute and chronic fat challenges, the failure of rMFGE8 to increase TG hydrolase activity in *Ces1d* KO enterocytes, and the lack of additive effects on enterocyte TG content and serum TG levels after olive oil gavage in double-KO mice for *Mfge8* and *Ces1d* (as compared with single-KO mice).

One potential limitation of our work is that genetic deletion of *Ces1d* may alter expression of other *Ces* enzymes that could contribute to the observed physiological effects. A recent profiling^26^ of the enzymatic activity of hydrolases in the murine small intestine identified multiple CES enzymes, including CES1D, and highlights the need to understand the function and regulation of the various intestinal hydrolases. Our data suggest that CES1D might be a rate-limiting enzyme in catabolism of cLDs from dietary lipids such that its deletion could impact the overall pathway, despite potential involvement of other CES enzymes.

Several of our observations strongly suggest that the MFGE8-integrin pathway uniquely regulates TG hydrolase activity relevant for catabolism of diet-derived cLDs. First, recombinant MFGE8 retains the ability to increase enterocyte TG hydrolysis in enterocyte KO for *Atgl*, the enzyme that regulates catabolism of cLDs derived from the basolateral circulation.^9^ Second, gavage of radio-labeled oleic acid in the setting of pretreatment (for the *Ces1d* KO and *HNFγ* KO studies) with the lipoprotein lipase inhibitor tyloxapol (which prevents breakdown of serum TG that is a prerequisite for absorption of fatty acids from the basolateral circulation) leads to accumulation of radiolabel in cLDs in the small intestine of *Mfge8* KO,^11^ *Ces1d* KO, and *HNFγ* KO mice. In contrast, intraperitoneal administration of [^3^H]oleic acid to *Mfge8* KO and WT mice resulted in a similar radioactive signal in total small intestinal lysates as well as in the cytosolic and microsomal fractions of small intestinal lysates. These data do not support a role for MFGE8 in regulating hydrolysis of basolateral-derived cLDs (which are present in the cytosolic fraction). The specificity of this pathway for luminal cLDs is consistent with our published work showing similar TG hydrolase activity in the liver and white adipose tissue of *Mfge8* KO mice as compared with WT mice.^11^

Our previous work identifies a biological program linking absorption of dietary fat with mobilization of fat stored in enterocyte cLDs for chylomicron production through MFGE8.^9–11^ Here, we delineate the molecular mechanisms coupling these two processes by showing that a dietary fatty acid (oleic acid), absorbed in part through MFGE8-dependent mechanisms, stabilizes protein levels and transcriptional activity of a nuclear hormone receptor, HNFγ, which then increases enterocyte CES enzyme expression and cLD hydrolysis. Both members of the HNF family of transcription factors (HNFα and HNFγ) constitutively bind fatty acids,^28^ with expression of HNFγ being predominately restricted to the small intestine.^15^

To explore the hypothesis that fatty acid uptake is an important step in MFGE8-induced increases in CES expression/activity, we focused on CD36, a fatty acid transporter with a well-established role in absorption of fatty acids in the proximal intestine.^38^ We had previously shown that in adipocytes and hepatocytes, MFGE8 induces cell surface translocation of CD36 leading to enhanced fatty acid uptake.^10^ In the intestine, we found markedly decreased expression of Cd36 in *Mfge8* KO enterocytes. Furthermore, *Cd36* KO enterocytes phenocopied *Mfge8* KO enterocytes with respect to HNFγ and CES1D protein expression, TG hydrolase activity, and differential gene expression profiles in enterocytes. Additionally, rMFGE8 failed to increase TG hydrolase activity in *Cd36* KO enterocytes. These data suggesting that fatty acid uptake regulates HNFγ-dependent transcription were further supported by the increase in HNFγ-dependent CES transcription and HNFγ protein levels induced by oleic acid. In sum, these data indicate to us that MFGE8-CD36-dependent uptake of dietary fats promotes enter-ocyte TG hydrolase activity by stabilizing HNFγ, leading to increased HNFγ-dependent transcription of CES enzymes. Our findings generate new questions related to the specific diet-derived fatty acids and/or metabolites that serve as ligands for HNF4γ, whether these fatty acids replace the constitutively bound fatty acid in HNFγ, and how this interplay regulates the transcriptional activity of HNFγ.

The MFGE8-integrin pathway has emerged as an interesting candidate for therapeutic targeting in metabolism. We have previously shown that MFGE8 promotes the development of obesity both through a direct effect on intestinal fat absorption^10^ and by reducing gastrointestinal motility, thereby allowing more time for nutrient absorption.^27^ Furthermore, these effects can be therapeutically targeted independent of each other given that they are mediated by different integrin receptors (αvβ5 for fat absorption and α8β1 for motility effects). More recently, we have shown that MFGE8 ligation of αvβ5 induces insulin resistance at the level of the insulin receptor and that blockade of this pathway leads to enhanced insulin sensitivity in the skeletal muscle and liver.^39^ Our work here identifies a carboxylesterase enzyme that is responsible for the effect of MFGE8 on catabolism of diet-derived cLDs and subsequent regulation of postprandial lipemia. This pathway can be targeted to reduce the severity of postprandial lipemia in obese, insulin-resistant patients while concurrently reducing fat absorption^10^ and enhancing peripheral tissue insulin sensitivity.^39^ Whether these benefits would outweigh the potential risks of targeting this biological pathway remains to be determined.

### Limitations of the study

(1) While the 3′ tag RNA-seq and ABPP-Mudpit tandem mass spectrometry data indicate altered expression and activity of CES1 hydrolases in *Mfge8* KO and *Hnf4γ* KO mice, the actual hydrolases identified do not always change in the same direction when comparing the sequencing and activity data. For instance, in *Hnf4γ* KO mouse enterocytes, Ces1f mRNA expression is downregulated but activity is increased. This raises the possibility of additional post-translational regulation of the hydrolases not addressed by our experimental design. (2) The accumulation of cytosolic lipid droplets in *Hnf4γ*KO mice could also be attributed to the effect of HNF4γ on lipid droplet synthesis, chylomicron formation, and/or chylomicron secretion. The present study does not address the impact of *Hnf4γ* on protein expression with roles in the aforementioned pathways. (3) Although the study has presented evidence for fatty-acid-dependent protein stabilization of HNF4γ, the mechanism of how fatty acid affects protein stability, the specific dietary fatty acid that serves as the ligand for HNF4γ, and the process that regulates transcriptional activity of HNF4γ in enterocytes after dietary lipid absorption have not been explored. (4) The potential therapeutic effects of the Ces1d deletion in enterocytes in the context of obesity and diabetes, specifically in regard to lowering postprandial lipemia, have not been studied and require further research.

## STAR★METHODS

### RESOURCE AVAILABILITY

#### Lead contact

Further information and requests for resources and reagents should be directed to and will be fulfilled by the lead contact, Kamran Atabai (Kamran.Atabai@ucsf.edu).

#### Materials availability

All mouse strains generated in this study are available from the lead contact upon request with a material transfer agreement.

#### Data and code availability

- 3′ Tag RNA sequencing data have been deposited at GEO database, MS data have been deposited at UCSD Mass Spectrometry Interactive Virtual Environment, and are publicly available as of the date of publication. Accession numbers are listed in the key resources table.
- This paper does not report original code.
- Any additional information required to reanalyze the data reported in this paper is available from the lead contact upon request.

### EXPERIMENTAL MODEL AND SUBJECT DETAILS

#### Mice

All animal experiments were approved by the UCSF Institutional Animal Care and Use Committee in adherence to NIH guidelines and policies. *Mfge8* KO mice were purchased from RIKEN and are in the C57BL/6 background and have been extensively characterized.^10,11^ *Ces1d* KO and *Ces1d* flox/flox have been well-characterized.^40–42^ *Villin-Cre* transgenic mice [Tg (Vil1 Cre) 997Gum)] (Jackson laboratories) were bred with *Ces1d* flox/flox mice to generate intestine-specific Ces1d KO (*Ces1d* int-KO) mice (*Ces1d* flox/flox Vil Cre+). *Ces1d* flox/flox Vil Cre negative mice were used as control. *Hnf4α* flox/flox Vil Cre ert2/*Hnf4γ*^Crispr^ mice are in a mixed background and have been characterized.^15^ We described Hnf4α flox/flox Hnf4γ^Crispr^ mice as *Hnf4γ* KO for our experiments. *Hnf4α* flox/ flox *Hnf4γ*+/+ mice were used as controls. Tg(TetO-Mfge8)^27^ transgenic mice containing the tetracycline-inducible Mfge8 construct were crossed with a *Mfge8* KO mice line created using a gene disruption vector and mice carrying the Tg(Vil-rtTA) transgene. *Cd36* KO mice has been extensively characterized.^43^ 6–10-week-old both male and female mice were used for the experiments.

#### Acute fat challenge

For acute fat challenge experiments, mice were fasted for 4 h and then subjected to an oral gavage of olive oil (200 μL). Mice were euthanized 2 h after the oil bolus and intestinal tissue pieces were collected for further experiments. Mice were treated IP with lipo-protein lipase inhibitor Tyloxapol (0.5 mg/g body weight of mice) 1 h before the oral gavage.^44^ WT mice were treated with FATP2 blocker Grassofermata (Cayman chemicals, catalog no. 26202) by IP injection at a dose of 300 mg/kg 2 h prior to oral gavage.^45^ WT mice were injected IP with either β5 blocking antibody (5 mg/kg) or isotype control antibody 2 h before acute fat challenge. Blood was drawn from mouse tail veins before and 2 h after oil gavage.

#### ^3^H oleic acid gavage

4 h-fasted mice were subjected to oral gavage with olive oil containing 5 μCi ^3^H-labeled oleic acid. Mice were treated IP with lipoprotein lipase inhibitor Tyloxapol (0.5 mg/g body weight of mice) 1 h before oral gavage. Prior to and 30, 60 and 120 min after olive oil/^3^H oleic acid administration, blood was collected from the tail vein. Mice were then euthanized and intestinal tissue pieces were procured and then freeze-dried in a tissue lyophilizer.

#### Injection of ^3^H oleic acid

4 h-fasted mice were subjected to intraperitoneal (I.P) injection of 10 μCi ^3^H-labeled oleic (PerkinElmer) acid in fat-free BSA. Mice were then euthanized and intestinal tissue pieces were procured.

#### Isolation of primary enterocytes

Primary enterocytes were harvested from intestinal jejunal segments.^11^ The jejunal lumen was washed with buffer A (115 mM NaCl, 5.4 mM KCl, 0.96 mM NaH_2_PO_4_, 26.19 mM NaHCO_3_, and 5.5 mM glucose buffer at pH 7.4, gassed for 30 min with 95% O2 and 5% CO_2_) and subsequently filled with buffer B (67.5 mM NaCl, 1.5 mM KCl, 0.96 mM NaH_2_PO_4_, 26.19 mM NaHCO_3_, 27 mM sodium cit-rate, and 5.5 mM glucose at pH 7.4, saturated with 95% O_2_ and 5% CO_2_) and incubated in buffer B for 15 min at 37 with constant shaking. After 15 min, the solution was discarded and the jejunal segments were transferred to a new 100 mm dish and filled with and incubated in buffer C (115 mM NaCl, 5.4 mM KCl, 0.96 mM NaH_2_PO_4_, 26.19 mM NaHCO_3_, 1.5 mM EDTA, 0.5 mM dithiothreitol, and 5.5 mM glucose at pH 7.4, saturated with 95% O_2_ and 5% CO_2_) for 15 min at 37°C with constant shaking after which the luminal contents were centrifuged and the pellet containing the epithelial cells used for subsequent experiments. The purity of the isolation was checks by FACS sorting cells using anti-Epcam antibody. Epcam-positive cells constituted 85–90% of the isolated cell pellet.

#### Caco-2 cell culture and treatment

Caco-2 cells are epithelial cells isolated from a 72-year-old, white, male with colorectal adenocarcinoma (ATCC-HTB-37). Cells were procured from ATCC. We have not authenticated the cell line. Caco-2 cells were differentiated for 3 weeks by maintaining confluent monolayer of cells in DMEM with 20%FBS at 37°C under 5% CO_2_. Cells were transfected with either Ces1 siRNA (Ambion, Catalog no. AM16708) or non-specific siRNA (Ambion, catalog no. AM4611) using Caco-2 cell transfection kit (Altogen biosystems, Catalog no. 6347) following manufacturers protocol. TG hydrolase activity was performed 2 days after siRNA transfection in cells serum-starved for 4 h.

#### HEK293 cell culture

HEK293 is a cell line exhibiting epithelial morphology that was isolated from the kidney of a human embryo (ATCC-CRL1573). Cells were procured from ATCC. We have not authenticated the cell line. Sex of the cells is unknown. Cells were cultured in DMEM with 10%FBS at 37°C under 5% CO_2_.

#### Human small intestine samples

Human intestinal epithelial cell lysates from small intestinal resection tissue samples of inflammatory bowel disease patients^46^ were provided by Dr. Rieder, Cleveland Clinic Foundation. These are de-identified human samples and therefore we do not have access to and cannot report the sex and/or gender of the subjects. N = 3 independent patient samples. Enterocytes were lysed using RIPA buffer followed by protein quantification by micro-BCA assay and 30 μg protein samples were loaded in 10% polyacrylamide gels for western blotting.

### METHOD DETAILS

#### TG hydrolase (TGH) activity assay

Protein was extracted from primary enterocytes in 100 mM potassium phosphate buffer by brief sonication. For Figure 5A, primary enterocytes were incubated with rMFGE8 or RGE proteins (10 μg/mL) in serum-free media for 1 h before proceeding with protein isolation. 60–100 μg protein was incubated with 100 μL TG substrate (25 nmol triolein/assay and 40,000 cpm/nmol ^14^C-triolein; PerkinElmer) and 35.5 μg mixed micelles of phosphatidylcholine and phosphatidylinositol (3:1, w/w), respectively, for 1 h at 37. After 1 h, the reaction was terminated by adding 3.25 mL methanol/chloroform/heptane (10:9:7, v/v/v) and 1 mL 100 mM potassium carbonate (pH 10.5 with boric acid). After centrifugation (800×g, 15 min, 4°C), radioactivity was measured in 1 mL of the upper phase by liquid scintillation counting.^11^ The radioactivity counts were normalized relative to protein concentration and the TG hydrolase activity was expressed as relative fold changes to the untreated control samples.

#### RNA isolation

RNA from primary enterocytes was isolated using Qiagen RNeasy plus micro kit. RNA from small intestinal tissues was isolated using Qiagen RNeasy lipid tissue mini kit.

#### 3′ tag RNA sequencing

Gene expression profiling of primary enterocyte RNA samples and total intestinal RNA samples were carried out using a 3′-tag-RNASeq protocol. Barcoded sequencing libraries were prepared using the QuantSeq FWD kit (Lexogen, Vienna, Austria) for multiplexed sequencing according to the recommendations of the manufacturer using the UDI-adapter and UMI Second-Strand Synthesis modules (Lexogen). High integrity total RNA samples were processed according to the QuantSeq default protocol. The fragment size distribution of the libraries was verified via micro-capillary gel electrophoresis on a LabChip GX system (PerkinElmer, Waltham, MA). The libraries were quantified by fluorometry on a Qubit fluorometer (LifeTechnologies, Carlsbad, CA), and pooled in equimolar ratios. The library pool was Exonuclease VII (NEB, Ipswich, MA) treated, SPRI-bead purified with KapaPure beads (Kapa Biosystems/Roche, Basel, Switzerland), quantified via qPCR with a Kapa Library Quant kit (Kapa Biosystems) on a QuantStudio 5 RT-PCR system (Applied Biosystems, Foster City, CA). Up to 48 libraries were sequenced per lane on a HiSeq 4000 sequencer (Illumina, San Diego, CA) with single-end 100 bp reads.

#### Bioinformatic analysis

FASTQ files were trimmed with Trimmomatic v0.38.1 (https://github.com/usadellab/Trimmomatic) and umi-tools (https://github.com/CGATOxford/UMI-tools) in order to remove low quality reads and any adapter contamination. The reads were mapped with HISAT2 v2.1.0 to the mouse genome (mm10/GRCm38). After mapping, all BAM files were used as input for HTSeq-count v0.91 to calculate transcript coverage. DESeq2 (v2.11.40)^47^ was used to find differentially expressed transcripts between samples for each sequencing depth. Differentially expressed genes (DEG) were determined based on whether the adjusted FDR was ≤0.05 and if a log2 fold-change of 0.5 or greater was observed. Data are deposited in the NCBI Gene Expression Omnibus (GEO) database under Accession no. GSE200320). Heatmaps (all significantly altered genes with FDR<0.05) were build using with GENE-E v3.0.215. In order to do pathway enrichment analysis, Ingenuity Pathways Analysis (IPA)^48^ was used focusing on differentially expressed genes with an FDR of <0.05. As determined from the DESeq2 differential gene expression analysis above. In order to predict transcription factors that regulate Ces family genes, iRegulon V1.3^49^ was run on the cytoscape 3.8.0 platform.^50^

#### Serine hydrolase probe MS experiments

For serine hydrolase probe MS experiments^13^ primary enterocytes were isolated from 5 mice to prepare a single sample (N = 1) and pooled cells together to extract protein by sonication in PBS. Protein concentrations were measured by micro-BCA or Bradford assay. 300–400 μg of protein at a concentration of 1 mg/mL was incubated with either Fluorophosphonate-biotin probe (final concentration of 5μM) or equivalent amount of DMSO (as negative control) for 60 min at 37°C. Excess probe was removed and protein precipitated with chloroform/methanol extraction by adding 2 volumes of methanol, 0.5 volume of chloroform and 1 volume of H_2_O and subsequently vortexed and centrifuged at 14,000 rpm for 5 min. The top layer was discarded and the protein layer collected from tube bottom. 2 volumes of methanol were added to the protein and stored it in −80°C overnight. The following day, the protein pellet was centrifuged, excess methanol removed and the protein pellet air dried for 15 min. The protein pellet was resuspended in freshly prepared 500μL 6M urea in 25 mM ammonium bicarbonate followed by the addition of 2.5 μL 1mM DTT and incubation at 65°C for 15 min. After cooling, 20μL 0.5M iodoacetamide was added to the protein which was then incubated at room temperature for 30 min to alkylate free cysteines. 70 μL of 10% SDS was added and heated for 5 min at 65°C. Samples were diluted with 3 mL PBS and incubated with 50 μL streptavidinagarose beads at room temperature for 2–3 h on a shaker. Beads were precipitated by centrifuging at 2500Xg for 2 min, washed, and resuspended in 250 μL 25 mM ammonium bicarbonate. 1 μg trypsin was added per sample and incubated overnight on a shaker at 37°C. Samples were then centrifuged and the supernatant containing peptides was collected followed by peptide desalting through C18 columns. Peptides were quantified and 200ng of sample loaded onto instrument for LC-MS analysis.

#### Mass spectrometry analysis

A nanoElute was attached in line to a timsTOF Pro equipped with a CaptiveSpray Source (Bruker, Hamburg, Germany). Chromatography was conducted at 40°C through a 25cm reversed-phase C18 column (PepSep) at a constant flowrate of 0.5 μL/min. Mobile phase A was 98/2/0.1% Water/MeCN/Formic Acid (v/v/v) and phase B was MeCN with 0.1% Formic Acid (v/v). During a 108 min method, peptides were separated by a 3-step linear gradient (5%–30% B over 90 min, 30%–35% B over 10 min, 35%–95% B over 4 min) followed by a 4 min isocratic flush at 95% for 4 min before washing and a return to low organic conditions. Experiments were run as data-dependent acquisitions with ion mobility activated in PASEF mode. MS and MS/MS spectra were collected with m/z 100 to 1700 and ions with z = +1 were excluded.

Raw data files were searched using PEAKS Online Xpro1.6 (Bioinformatics Solutions Inc., Waterloo, ON, Canada). The precursor mass error tolerance and fragment mass error tolerance were set to 20 ppm and 0.03 respectively. The trypsin digest mode was set to semi-specific and missed cleavages was set to 2. mouse Swiss-Prot reviewed (canonical) database (downloaded from UniProt) and the common repository of adventitious proteins (cRAP, downloaded from The Global Proteome Machine Organization) totaling 20,487 entries were used. Carbamidomethylation was selected as a fixed modification. Oxidation (M) was selected as a variable modification.

Experiments were performed in biological triplicate. Resulting combined datasets were subjected to the following filtration criteria:

1. Database Search (−10log(p value) R 20, 1% peptide and protein FDR).
2. Cross-reference with a serine hydrolase proteome dataset.
3. Generate ratio of Probe/No Probe. Require ≥R2 Unique Peptides and ≥R3 peptides total with probe treatment.
4. Proteins determined to be probe enriched were 3-fold more detected in Probe-treated sample compared to No Probe (ratio of ≥R3).

Data is available via the UCSD Mass Spectrometry Interactive Virtual Environment, a full member of the Proteome Exchange consortium, under the dataset number (Accession no. MSV000089304).

#### Protein isolation and Western blot

Primary enterocytes were centrifuged in PBS and the cell pellet was incubated with protein lysis buffer (20mM Tris-HCl pH8.0, 137mM NaCl, 1% Nonidet P-40 (NP-40) and 2mM EDTA) overnight in −80°C before protein isolation was carried out by repeated cycles of freezing and thawing. To isolate protein from tissue samples, a TissueLyser (Qiagen) was used to homogenize the tissue samples in lysis buffer. Lysates were then centrifuged at 13,000×g for 10 min at 4°C to pellet debris and supernatants were stored in −80°C for future use. Protein concentration was measured by Bradford assay, followed by western blotting using standard procedure. 10–20 μg protein samples in SDS-PAGE were resolved in 7.5%–10% gels (Bio-Rad) and transblotted onto polyvinylidene fluoride membranes (Millipore). Membranes were blocked with 5% BSA-PBST for 1 h and then incubated with primary antibody (listed in Table S1) overnight at 4°C. Membranes were then washed in 0.15% PBST 3–5 times at 5 min per wash before incubation with HRP-conjugated secondary antibodies for 1 h. Membranes were washed 3–5 times in 0.15% TBST. Immunoreactive bands were generated using an Immobilon Western chemiluminescence HRP-conjugated substrate (Amersham) and developed either on a film (Kodak) or imaged in a ChemiDoc. When Li-Cor secondary antibodies were used to generate bands, membranes were imaged in Li-Cor Odyssey. Membranes were subsequently deprobed using Restore Western blot stripping buffer (Thermo scientific) and re-probed using other primary antibodies

#### Immunofluorescence staining

Mice jejunal tissues were fixed in 4% Z-fix overnight followed by cryopreservation in 15% and then 30% sucrose in PBS. Tissues were then embedded in OCT medium and cryo-sectioned (30 μm) on frost-free slides using a cryo-stat. Coverslips/slides were then washed with PBST (0.5%Tween or Triton X-100) and incubated in blocking buffer (PBST, 1% BSA and 5% donkey serum) for 1 h. Tissue sections were incubated overnight with the primary antibody against Epcam (primary antibodies and their dilutions are listed in Table S1) in blocking buffer at 4°C. On the following day, tissue sections were washed with PBST 3 times and then incubated with the secondary antibodies (donkey anti rat Alexafluor in 1:100 dilution) for 1 h, washed, stained with Bodipy 493/503 (2 mg/mL) for 30 min followed by mounting in Vectashield (H-1200) DAPI. For staining for active hydrolases, the fixed tissues on slides were preincubated for 20 min with assay buffer (50 mM Tris-HCl, pH 7.4; 1 mM EDTA; 100 mM NaCl; 5 mM MgCl2 and 0.1% (w/v) BSA) followed by incubation for 60 min with TAMRA-FP in the assay buffer (0.5 μM final concentration).^12^ Slides were then washed 3 times in 0.1M phosphate buffer before mounting with DAPI. Images were captured in the confocal microscope Zeiss LSM 780-FLIM and processed in ImageJ.

#### Microsome isolation

A microsome isolation kit (ThermoFisher Scientific) was used for separating cytosol and microsome from jejunal tissues following the manufacturer’s protocol. Jejunal tissues from mice fed with radiolabeled oleic acid were resuspended in 200 μL of olive oil. 50 mg tissue was homogenized in homogenizing buffer, incubated on ice for 1 h and centrifuged at 10,000g for 10 min to clear debris. The supernatant was centrifuged at 20,000g for 20 min in the pellet containing the microsomes, washed and resuspended in resuspension buffer with the supernatant representing the cytosolic fraction. Radioactivity was measured in each of these fractions using liquid scintillation counting.

#### Incorporation of ^3^H radiolabel in intestinal triglycerides

After extracting lipids from the intestinal tissue pieces, we resolved lipids in hexane/isopropyl ether/glacial acetic acid 60:40:4 (v/v/v) by thin-layer chromatography.^11^ Lipid species were identified according to standards, and bands corresponding to the triglyceride were scraped, incubated overnight in scintillation liquid, and quantified in scintillation counter.

#### Chronic high-fat feeding

8-week-old male mice were placed on high-fat diet (60%kcal% fat, Research Diet Inc, catalog no. d12492) for 3 weeks after which they were fasted for 12 h before evaluation of the serum and intestinal TG content.

#### Serum and intestinal TG measurement

Serum and intestinal TG content was measured using TG measurement kit (Cayman Chemical) following manufacturers’ protocol. Blood was collected from mouse tail veins and serum isolated by centrifuging blood samples for 15 min at 2000×g. 5 μL serum were used to measure TG content. For measuring TG content in the intestinal samples, approximately 50 mg tissue were homogenized in lysis buffer (supplied with the kit) and used 10 μL of the tissue lysate to measure TG content. The total TG content were normalized to the weight of the tissue.

#### Ex-vivo overexpression of HNF4γ

An adenoviral expression vector containing mouse *Hnf4γ* gene (pAV[EXP]-EGFP CMV>mHnf4g (NM_013920.2), referred to here as AD-*Hnf4γ*-GFP) was obtained from Vector Biolabs. The intestinal lumen was flushed with PBS and pieces of small intestine from *Hnf4γ* KO mice were incubated with either blank adenoviral vector or *Hnf4γ*-OV for 24 h in DMEM. Intestinal pieces were then washed with PBS and lysed for protein extraction and Western blot.

#### Luciferase assay

The 500-bp region of the putative promoter/enhancer regions of *Ces1d* gene (corresponding to the ChIP-Seq peak, accession no. GSE112946)^15^ were subcloned into a luciferase vector with a minimal promoter (PGL4) and dual luciferase activity assay was performed in control and HNF4γ-overexpressed HEK293 cells in the presence and absence of oleic acid. HEK293 cells were plated on 96-well plates and infected with Hnf4γ-expressing adenovirus for 6 h in serum-free media and then cells were incubated with complete media overnight. On the following day, Hnf4γ-adenovirus infected cells were transfected with 0.5 μg of reporter plasmid carrying the firefly luciferase gene under the control of Ces gene promoters containing the HNF4γ binding sequences in PGL-4 vector, and 0.5 μg of reference plasmid pRL-TK carrying the Renilla luciferase gene under the control of the simian virus 40 enhancer and promoter (Promega). Lipofectamine 3000 reagent (Invitrogen) was used as a transfection reagent following the manufacturer’s protocol. After 24 h, cells were treated with oleic acid (0.6 μM final concentration) for another 12 h. Cells were lysed in 200 μL of passive lysis buffer (Promega). Firefly luciferase and Renilla luciferase activities were measured using Dual-Luciferase reporter assay system (Promega) on a 96-well plate on a plate reader. Relative activity was defined as the ratio of firefly luciferase activity to Renilla luciferase activity.

#### HNF4γ protein stability assay

HEK293 cells were infected with Hnf4γ-adenovirus for 6 h in serum-free media and then the cells were incubated with complete media overnight. On the following day, the cells were first treated with cyclohexamide (100 μg/mL) followed by treatment with oleic acid in fat-free BSA or BSA alone. Cells were then lysed 6, 12 and 24 h after treatment. Protein samples were used to detect HNF4γ protein level by western blotting.

#### Recombinant MFGE8 (rMFGE8) treatment

rMFGE8 and RGE constructs consisted of murine cDNA of *Mfge8* (long isoform) fused with the human FC domain. They were expressed in High Five cells and affinity purified.^10,39^ The RGE construct contains a point mutation that changes the integrin-binding RGD sequence to RGE. Primary enterocytes were treated with either RGE or rMFGE8 (10 mg/mL) for 1 h and then washed in PBS and processed for TG hydrolase activity assay.

### QUANTIFICATION AND STATISTICAL ANALYSIS

Unpaired t test was used when comparing two groups. Multiple groups were compared using One-way ANOVA. Further pairwise comparisons were performed using BonFerroni’s posttest. For analysis of postprandial serum level of ^3^H at different timepoints after ^3^H-labeled oleic acid gavage in WT and knock out mice were compared using two-way ANOVA followed by BonFerroni’s posttest. All statistical analysis was performed using GraphPad Prism 9.4.1. Data are represented as mean ± SEM. p < 0.05 were considered significant. Total number of samples (N) per experiment and number of independent experiments for each data panel have been provided in respective figure legends.

## Supplementary Material

1

## Figures and Tables

**Table:** KEY RESOURCES TABLE

REAGENT or RESOURCE	SOURCE	IDENTIFIER
Antibodies		
CES1D	Santacruz biotechnology	sc-374160; RRID:AB_10988772
Human CES1	R & D biosystems	AF 4920; RRID:AB_2291518
Human CES2	R & D biosystems	AF 5657; RRID:AB_10584991
HNF4γ	Proteintech	25801–1-1AP; RRID:AB_2880242
MFGE8	R & D biosystems	AF2805; RRID:AB_2281868
HSP90	Santacruz biotechnology	sc-7947; RRID:AB_2121235
GAPDH	Cell signaling technology	2118; RRID:AB_561053
EPCAM (CD326)	BD pharmingen,	552370; RRID:AB_394370
Anti-rabbit IgG, HRP-linked Antibody	Cell signaling technology	7074; RRID:AB_2099233
Anti-mouse IgG, HRP-linked Antibody	Cell signaling technology	7076; RRID:AB_330924
Donkey anti rat Alexa Fluor 594 secondary antibody	ThermoFisher Scientific	A21209; RRID:AB_2535795
Bacterial and virus strains		
pAV[EXP]-EGFP CMV>mHnf4g (NM_013920.2)	Vector biolabs	NA
Biological samples		
Human intestinal epithelial cell lysates from small intestinal resection tissue samples of inflammatory bowel disease patients	Cleveland clinic	NA
Chemicals, peptides, and recombinant proteins		
^14^C-triolein	PerkinElmer	Catalog no. NEC674050UC
^3^H-Oleic acid	PerkinElmer	Catalog no. NET289001MC
Phosphatidylcholine	Sigma Aldrich	Catalog no. P3556
Phosphatidylinositol	Sigma Aldrich	Catalog no. P6636
ActivX TAMRA-FP serine hydrolase probe	ThermoFisher Scientific	Catalog no. 88318
ActivX^™^ Desthiobiotin-FP Serine Hydrolase Probe	ThermoFisher Scientific	Catalog no. 88317
Bodipy 493/503	ThermoFisher Scientific	D3922
Vectashield with DAPI	Vector laboratories	H-1200–10
Grassofermata	Cayman chemicals	catalog no. 26202
high-fat diet (60%kcal% fat)	Research Diet Inc	catalog no. d12492
Critical commercial assays		
Qiagen RNeasy plus micro kit	Qiagen	catalog no. 74034
Qiagen RNeasy lipid tissue mini kit	Qiagen	catalog no. 74804
Microsome isolation kit	Sigma-Aldrich	catalog no. MAK-340
TG colorimetric assay kit	Cayman Chemical	catalog no. 10010303
Dual luciferase reporter assay system	Promega corp	catalog no. E1910
Caco-2 cell transfection kit	Altogen biosystems	Catalog no. 6347
Deposited data		
3’ Tag RNA sequencing data	This paper	GEO Accession no. GSE200320
Mass spectrometry data	This paper	Accession no. MassIVE: MSV000089304
Experimental models: Cell lines		
Caco-2 cell line	ATCC	ATCC-HTB-37
HEK293	ATCC	CRL-1573
Experimental models: Organisms/strains		
Tg (Vil1 Cre) 997Gum)	Jackson laboratories	NA
*Ces1d* KO	Ref. Lian et al.^40 41^	NA
*Ces1d* flox/flox	Ref. Lian et al.^42^	NA
*Hnf4α* flox/flox Vil Cre ert2/*Hnf4*γ^Crispr^	Ref. Chen et al.^15^	NA
*Mfge8*KO	Ref. Khalifeh-Soltani etal.^10,11^	NA
Tg(TetO-Mfge8)	Ref. Khalifeh-Soltani et al.^27^	NA
*Cd36* KO	Ref. Cifarelli et al.^43^	NA
Oligonucleotides		
Ces1 siRNA	Ambion	Catalog no. AM16708
Non-specific siRNA	Ambion	Catalog no. AM4611
Recombinant DNA		
pGL4.23[luc2/minP]	Promega	E8411
Software and algorithms		
Graphpad Prism 9	Graphpad	Prism - GraphPad
Fiji ImageJ	ImageJ	Fiji (imagej.net)
Ingenuity Pathway Analysis (IPA)	Qiagen IPA	QIAGEN Ingenuity Pathway Analysis (IPA)

## Highlights

- MFGE8 links dietary lipid uptake with hydrolysis of enterocyte lipid droplets (cLDs)
- MFGE8-dependent dietary fatty acid uptake stabilizes HNF4γ protein in enterocytes
- HNF4γ regulates transcription of carboxylesterase 1 (Ces1) hydrolases
- CES1D regulates postprandial lipemia through hydrolysis of diet-derived cLDs
