# Supplementary material for: MFGE8 links absorption of dietary fatty acids with catabolism of enterocyte lipid stores through HNF4γ-dependent transcription of CES enzymes
Source: Cell Rep. Author manuscript; Available in PMC 2023 Apr 27. (PMC10138282; doi:10.1016/j.celrep.2023.112249)
Supplement: 1 [file NIHMS1887305-supplement-1.pdf]

Cell Reports, Volume *42*

Supplemental information

MFGE8 links absorption of dietary fatty acids with catabolism of enterocyte lipid stores through HNF4γ-dependent transcription of CES enzymes

Ritwik Datta, Mohammad A. Gholampour, Christopher D. Yang, Regan Volk, Sinan Lin, Michael J. Podolsky, Thomas Arnold, Florian Rieder, Balyn W. Zaro, Michael Verzi, Richard Lehner, Nada Abumrad, Carlos O. Lizama, and Kamran Atabai

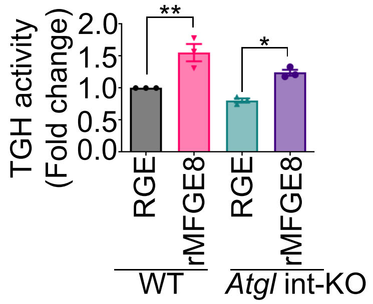

Supplementary Figure 1: MFGE8 regulates enterocyte TG hydrolase activity independent of ATGL (related to Figure 1). TG hydrolase activity in WT and Atgl int-KO primary enterocytes after treatment with rMFGE8 or RGE control for 1 hour. N=3 independent experiments. Data expressed as Mean ± S.E.M. *P < 0.05, **P < 0.01. Data were analyzed by one-way ANOVA followed by Bonferroni's posttest.

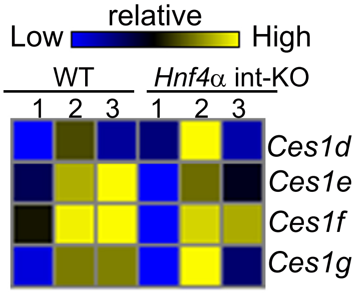

Supplementary figure 2: Unchanged expression of *Ces1* genes in *Hnf4*α int-KO mouse enterocytes (related to Figure 2). Heatmap generated from previously published RNA sequencing data (Accession no. GSE 200320) from enterocytes of WT and intestine-specific *Hnf4*αKO mice showing unchanged expression of the Ces1 genes.

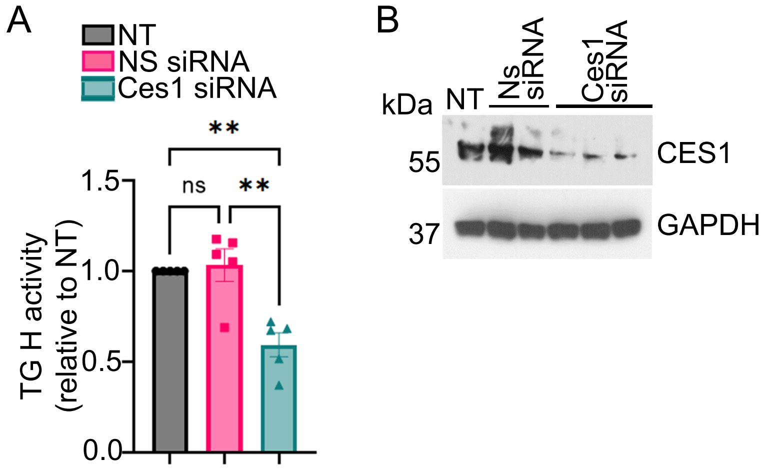

Supplementary figure 3: Si-RNA-mediated knockdown of Ces1 gene dampens TG hydrolase activity in Caco-2 cells (related to Figure 3). (A)TG hydrolase activity in untreated (NT), non-specific siRNA-treated (NS siRNA) and Ces1 siRNA -treated Caco-2 cells. N=5 independent experiments. Data expressed as mean ± S.E.M. **P < 0.01. Data were analyzed by one-way ANOVA followed by Bonferroni's posttest. (B) Western blot showing CES1 protein level in Caco-2 cell lysates after siRNA treatments.

tacatctcatacattgcagacttgggaacacagaaaggttatttgatttggacaccagccttaagctatggtcactg
tggtcaccattcaaatcacactaatgcacagcaaggctataacctctctctctctctctctctctctctctctct
ctctctctctccctctctttcaaacaacccacagctcctgccaggaatataatgtaacatggtaccgcactgtgtttctc
atttctcacatcctcccaagatgctcaaactg**agaacattgccatgg**aa**acagctcaaaggtca**aagttattgatct
acttcccctgctcttatcacctatgcctgaatgaacataaagctcttgtatttcttcatttcatcccaagtaactcc
tcactggttaaactagatttttcccacaagtaagtgattatctgatgagacagacagacactaccctgatggggcac
cttccaaatgtgctttctttctgcacacaaaaatccaccatgttgctaatcctatctcatacttgctttctcctgaaagtc
ttcagtcttctgctagtgctatatctaaacctattaagtaccaggtactctaattgacttgagcttgaaaggaaatata
atttttcttctttttaatttgaagagtatgtcaataagggaaggaatgagagtgtgtaa

Supplementary figure 4: HNF4γ binds to enhancer region of Ces1d gene (related to Figure 3).
DNA sequence of the HNF4γ-binding region of Ces1d enhancer as analyzed from previously published chip sequencing data (Accession no. GSE 112946). DNA motifs that bind HNF4γ are highlighted in red.

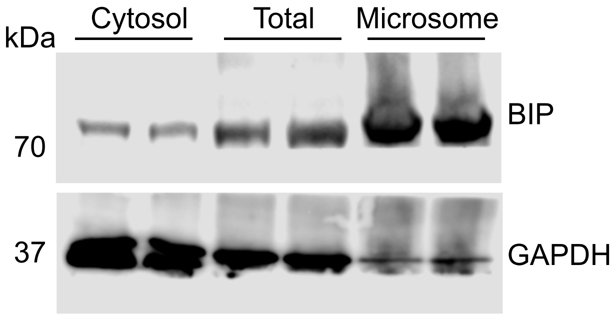

Supplementary Figure 5: Validation of cytosolic and microsomal fractionation from total intestinal lysate (related to Figure 3). Western blot showing relative enrichment of microsomal (BIP) and cytosolic marker (GAPDH) proteins in respective subcellular fractions.

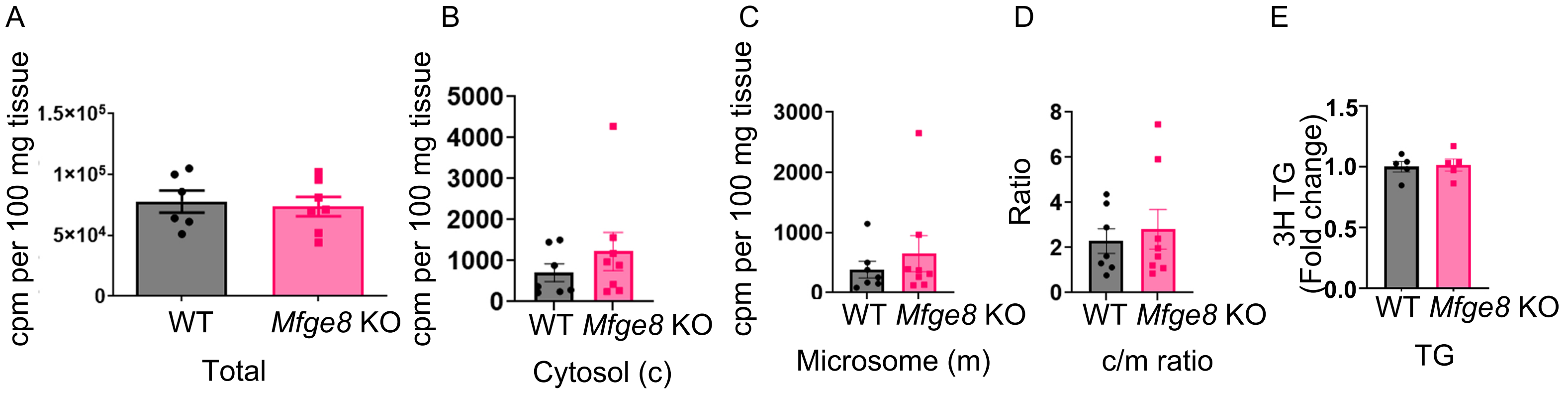

Supplementary Figure 6: MFGE8 does not impact hydrolysis of cLDs derived from the basolateral surface (related to Figure 6). (A-C) 3H signal in the total (A), cytosolic fraction (B), microsomal fraction (C), and the ratio of cytosolic to microsomal radioactive signal (D) and 3H signal in the TGs separated by TLC (E) in the small intestines from WT and Mfge8 KO mice 2 hours after IP injection of 3H-labeled oleic acid. N=6-8 mice in each group for panels A-D and N=5 mice in each group for panel E. Data merged from 2 independent experiments. A mix of 5-7-week-old male and female mice were used for these experiments. Data expressed as Mean ± S.E.M. Data were analyzed by unpaired student's t-test.

Antibody	Make and Catalog no.	Host species	Application and dilution
CES1D	Santacruz biotechnology, sc-374160	Mouse	WB (1:400)
Human CES1	R & D, AF 4920	Goat	WB (1:500)
Human CES2	R & D, AF 5657	Goat	WB (1:1000)
HNF4γ	Proteintech, 25802-1AP	Rabbit	WB (1:500)
MFGE8	R & D, AF2805	Goat	WB (1:500)
HSP90	Santacruz biotechnology, sc-7947	Rabbit	WB (1:500)
GAPDH	Cell signaling technology, 2118	Rabbit	WB (1:2000)
EPCAM (CD326)	BD pharmingen, 552370	Rat	IF (1:200)

Supplementary Table 1: List of primary antibodies (related to Star Methods).
